# Spatial Transcriptomics Reveals Transcriptomic and Immune Microenvironment Reprogramming during Thyroid Carcinoma Dedifferentiation

**DOI:** 10.1002/advs.202506925

**Published:** 2025-09-04

**Authors:** Kang Ning, Bu Zou, Yongchao Yu, Taonong Cai, Zhenyu Luo, Yu Guo, Yi Wu, Xiujiao Shen, Hao Li, Mengyuan Fang, Jian Bu, Han Hong, Zan Jiao, Tong Wu, Yulong Wang, Tianrun Liu, Weichao Chen, Wanming Hu, Mingjie Jiang, Ankui Yang

**Affiliations:** ^1^ Department of Head and Neck Surgery Sun Yat‐sen University Cancer Center Guangzhou 510060 China; ^2^ State Key Laboratory of Oncology in Southern China Guangzhou 510060 China; ^3^ Collaborative Innovation Center for Cancer Medicine Guangzhou 510060 China; ^4^ Department of Thyroid Surgery Sun Yat‐sen Memorial Hospital Sun Yat‐sen University Guangzhou 510220 China; ^5^ State Key Laboratory of Ophthalmology Zhongshan Ophthalmic Center Sun Yat‐sen University Guangdong Provincial Key Laboratory of Ophthalmology and Visual Science Guangdong 510060 China; ^6^ Department of Pathology Sun Yat‐sen University Cancer Center Guangzhou 510060 China; ^7^ The Second Clinical College of Hainan Medical University Haikou 570216 China; ^8^ Department of Ultrasound Changsha Hospital for Maternal & Child Health Care Affiliated to Hunan Normal University Changsha 410007 China; ^9^ Zhongshan School of Medicine Sun Yat‐sen University Guangzhou 510030 China; ^10^ Department of Head and Neck Surgery Fudan University Shanghai Cancer Center Shanghai 200003 China

**Keywords:** anaplastic thyroid carcinoma, differentiated thyroid carcinoma, M2 macrophage, PDCD4, spatial transcriptomics

## Abstract

Anaplastic thyroid carcinoma (ATC) is one of the most lethal human malignancies, often evolving from differentiated thyroid carcinoma (DTC) through a poorly understood dedifferentiation process. To elucidate this transition, spatial transcriptomic sequencing (spRNAseq) is performed on seven samples containing coexisting regions of ATC, poorly differentiated thyroid carcinoma, and DTC. SpRNAseq revealed that ATC regions were characterized by upregulated genes involved in immune suppression, angiogenesis, and extracellular matrix remodeling. Whole‐exome sequencing and inferCNV analysis confirmed that adjacent DTC regions harbored mutational burdens comparable to those of ATC regions, suggesting early genomic priming for dedifferentiation. Trajectory analysis delineated a stepwise reprogramming process and identified four gene modules associated with the loss of thyroid differentiation, among which PDCD4 and TYMP emerged as key regulators. Notably, TYMP⁺ tumor‐associated macrophages (TAMs) were highly enriched in ATC regions and contribute to an immunosuppressive microenvironment. Mechanistic experiments demonstrated that loss of PDCD4 led to eIF4A‐dependent overexpression of immunosuppressive effectors, promoting the high infiltration of TYMP⁺TAMs in ATC. These findings support that coexisting DTC regions with ATC‐like genomic alterations undergo sequential transcriptomic reprogramming and immune microenvironment remodeling to evolve into a full ATC pathological phenotype, in which PDCD4 loss–induced TAMs formation plays a critical role.

## Graphical abstract

Spatial transcriptomic analysis of Anaplastic thyroid carcinoma (ATC)‐differentiated thyroid carcinoma (DTC) coexisting tumors reveals that coexisting DTC regions with ATC‐like genomic alterations undergo sequential transcriptomic reprogramming and immune microenvironment remodeling, ultimately evolving into a full ATC pathological phenotype. Notably, PDCD4 loss drives eIF4A‐dependent upregulation of immunosuppressive effectors, promoting TYMP⁺ tumor‐associated macrophages enrichment, which represents a key mechanism in establishing the immunosuppressive microenvironment of ATC.

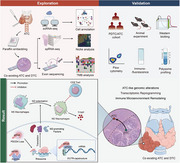

## 1 Introduction

Anaplastic thyroid carcinoma (ATC) is a highly aggressive malignancy originating from thyroid follicular epithelial cells, characterized histologically by marked cellular pleomorphism, nuclear atypia, extensive necrosis, and complete loss of thyroid‐specific differentiation markers.^[^ ^1^ ^]^ Although ATC accounts for only 1–2% of all thyroid cancers, its clinical course is extremely aggressive, often presenting with rapid local invasion and distant metastasis within a short time frame, resulting in a dismal prognosis.^[^ ^2^, ^3^ ^]^ In stark contrast to the favorable therapeutic responsiveness and long‐term survival observed in differentiated thyroid carcinoma (DTC), the median overall survival for patients with ATC is approximately five months, with a one‐year survival rate below 20% and a five‐year survival rate of less than 5%.^[^ ^4^, ^5^ ^]^ Current therapeutic strategies largely rely on multimodal approaches, including surgical resection, radiation therapy, and chemotherapy; however, clinical outcomes remain unsatisfactory.^[^ ^3^, ^6^ ^]^

From a molecular and pathological perspective, ATC and DTC represent two ends of the thyroid cancer differentiation spectrum.^[^ ^7^ ^]^ Approximately 50% of ATC specimens contain residual DTC components, suggesting that ATC may arise from a stepwise dedifferentiation process of preexisting DTC.^[^ ^8^ ^]^ This transition is often accompanied by the accumulation of key driver mutations, including *TP53* inactivation, *TERT* promoter activation, and aberrant activation of oncogenic pathways such as *BRAF* and *PIK3CA*.^[^ ^9^, ^10^, ^11^ ^]^ These genetic alterations collectively promote aggressive phenotypes characterized by uncontrolled proliferation, resistance to apoptosis, enhanced invasiveness, and immune evasion. However, most current studies on ATC are based on non‐matched, cross‐sectional samples or focused solely on genomic alterations, limiting our understanding of the temporal and spatial dynamics of transcriptomic, proteomic, and immune changes during DTC‐to‐ATC progression.^[^ ^7^, ^12^, ^13^, ^14^ ^]^

The tumor immune microenvironment (TIME), a complex ecosystem composed of immune cells, stromal elements, inflammatory mediators, and metabolic regulators, plays a central role in tumor initiation, progression, immune evasion, and response to therapy.^[^ ^15^ ^]^ Compared to DTC, ATC exhibits a profoundly immunosuppressive TIME, characterized by enrichment of tumor‐associated macrophages (TAMs), tumor‐associated fibroblasts, and functionally exhausted CD8⁺ T cells.^[^ ^7^, ^16^, ^17^ ^]^ Recent advances in spatial RNA sequencing (spRNAseq) have provided powerful tools to delineate region‐specific transcriptional landscapes within intact tissue architecture.^[^ ^18^ ^]^ This approach is particularly well‐suited to analyzing tumors harboring both DTC and ATC components within the same specimen, enabling the dissection of spatially resolved molecular alterations and TIME remodeling during dedifferentiation.

In this study, spRNAseq was employed to perform paired analysis on seven tumor samples containing both DTC and poorly differentiated thyroid carcinoma (PDTC)/ATC regions. A pseudotemporal trajectory was constructed to depict the transcriptomic dynamics during the dedifferentiation from DTC to ATC. Furthermore, in‐depth analysis of the TIME and key regulatory genes across distinct differentiation states revealed potential therapeutic strategies aimed at reprogramming the immunosuppressive TIME of ATC.

## 2 Result

### 2.1 Single‐Sample spRNAseq Analysis of ATC/PDTC‐DTC Coexisting Samples

**Figure 1:**
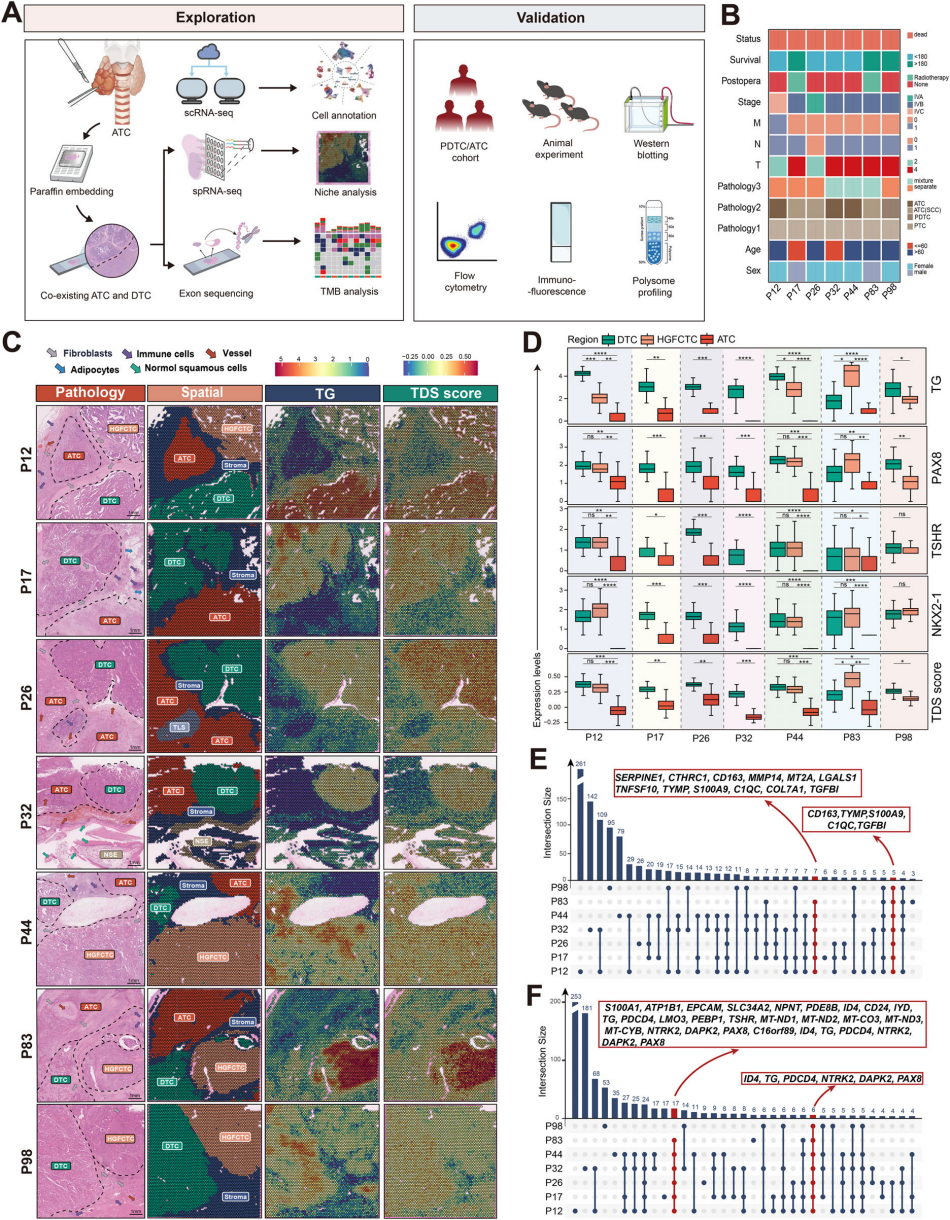
SpRNAseq analysis of individual samples in ATC/PDTC‐DTC coexisting samples. A) Workflow of spRNAseq for samples with coexisting ATC/PDTC and DTC. B) Clinical characteristics of the seven patients included for spRNAseq. C) Spatial distribution of histopathological annotations, TSD scores, and TG gene expression in seven ATC/PDTC–DTC coexisting samples. D) Boxplots showing the expression levels of TDS score and thyroid differentiation‐related genes in each sample. E–F) UpSet plots illustrating the overlap of upregulated (E) and downregulated (F) DEGs between the ATC/PDTC and coexisting DTC regions across individual samples. **ATC**: Anaplastic Thyroid Carcinoma; **DEGs**: Differentially Expressed Genes; **DTC**: Differentiated Thyroid Carcinoma; **HGFCTC**: High‐grade Follicular Cell‐derived Thyroid Carcinoma; **PDTC**: Poorly Differentiated Thyroid Carcinoma; **spRNAseq**: Spatial RNA Sequencing; **TDS**: Thyroid Differentiation Score; **TLS**: Tertiary Lymphoid Structures; **NSE**: Normal Squamous Epithelium. Significance in D was determined using one‐way ANOVA and Bonferroni multiple comparison test ^*^ *p* < 0.05, ^**^ *p* < 0.01, ^***^ *p* < 0.001, ^****^ *p* < 0.0001.

Prior to spRNAseq, all ATC/PDTC surgical specimens underwent Hematoxylin‐Eosin (HE) staining review and quality control by experienced pathologists. Ultimately, seven thyroid carcinoma samples containing regions with varying differentiation status were included for analysis (Figure S1–S2, Supporting Information). This within‐patient design enabled direct comparison of transcriptomic profiles between regions of different differentiation, minimizing inter‐individual heterogeneity. Key findings were further validated using publicly available thyroid cancer single‐cell RNA sequencing (scRNAseq) data, tissue‐based assays, and both in vitro and in vivo experiments (**Figure** **1**A). Among the seven cases, one was diagnosed as PDTC and three as thyroid squamous cell carcinoma (Figure 1B; Table S1, Supporting Information). All patients eventually succumbed to the disease, with a median overall survival of 254 days (range: 37–829). Based on the 2022 WHO classification,^[^ ^19^ ^]^ spRNAseq regions were annotated by pathologists as ATC, high‐grade follicular cell‐derived thyroid carcinoma (HGFCTC), DTC, and stroma (Figure 1C). HGFCTC, encompassing PDTC and differentiated high‐grade thyroid carcinoma (DHGTC), is considered an intermediate state between DTC and ATC.^[^ ^19^ ^]^ Notably, P26 exhibited prominent tertiary lymphoid structures (TLS), while P32 included areas of normal squamous epithelium (NSE).

We further assessed the thyroid differentiation score (TDS) and key thyroid lineage markers (*TG*, *PAX8*, *TSHR*, *NKX2‐1*).^[^ ^20^ ^]^ These markers showed significant differences between ATC and non‐ATC regions, while differences between DTC and HGFCTC were less pronounced (Figure 1C,D; Figure S3A,B, Supporting Information). Spatial transcriptomic gradient (STG) analysis revealed a clear gene expression gradient across differentiation states, and genes enriched in the transition from well‐ to poorly differentiated regions were associated with epithelial–mesenchymal transition, complement activation, and hypoxia (Figure S4A,B, Supporting Information). Differential expression and pathway analyses of high versus low differentiation regions in each sample showed that genes upregulated in well‐differentiated regions were mainly involved in thyroid hormone metabolism and epithelial differentiation, while poorly differentiated regions were enriched for genes related to chemotaxis, myeloid cell migration, and collagen fibril organization (Figure S4C–E and Table S2, Supporting Information). An upset plot summarized commonly differentially expressed genes (DEGs) across patients, identifying five consistently upregulated genes (*CD163*, *TYMP*, *S100A9*, *C1QC*, *TGFBI*) and six downregulated ones (*ID4*, *TG*, *PDCD4*, *NTRK2*, *DAPK2*, *PAX8*) (Figure 1E,F). These genes are primarily associated with myeloid infiltration, cell death, and angiogenesis.

### 2.2 Integrated Multi‐Sample spRNAseq Reveals Tumor Heterogeneity

**Figure 2:**
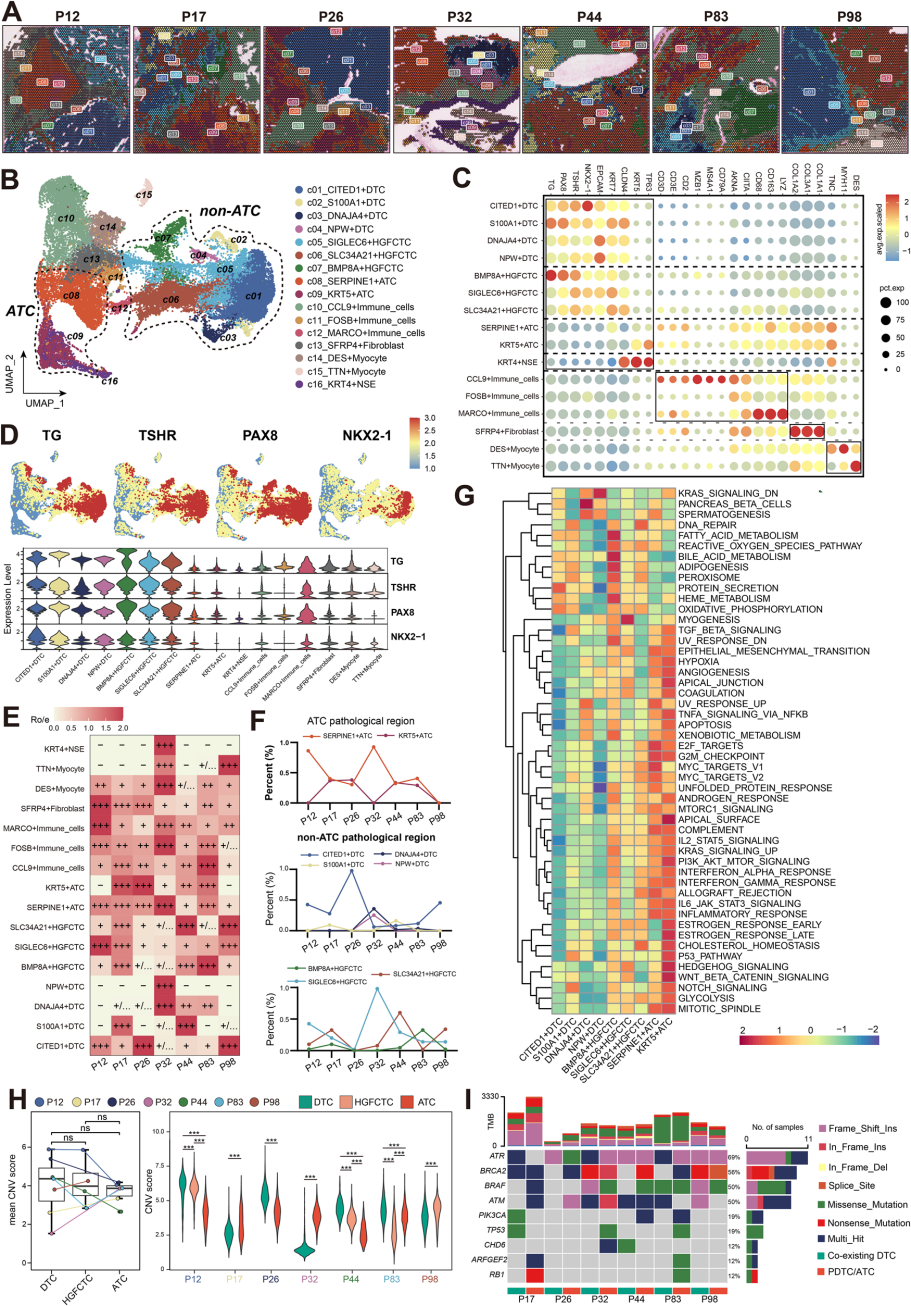
Multi‐sample integration and tumor heterogeneity analysis of ATC/PDTC‐DTC coexisting samples. A) Spatial distribution of each niche following multi‐sample integration analysis. B) UMAP plot showing the annotated niches after multi‐sample integration analysis. C) Dot plot displaying marker gene expression across the annotated niches. D) Distribution of thyroid differentiation‐related genes among different tumor niches. E) Ro/e plot showing the relative abundance of each niche across individual samples. F) Line chart illustrating the proportion of different tumor niches within different histopathological regions. G) GSVA‐based pathway analysis revealing tumor‐related gene expression patterns across different tumor niches. H) Boxplot (mean expression) and violin (single‐sample) plot comparing inferCNV scores among different tumor niches and pathological regions. I) Waterfall plot summarizing mutations in thyroid cancer–associated genes and TMB from WES of ATC‐DTC coexisting samples. **ATC**: Anaplastic Thyroid Carcinoma; **DTC**: Differentiated Thyroid Carcinoma; **HGFCTC**: High‐Grade Follicular Cell‐derived Thyroid Carcinoma; **PDTC**: Poorly Differentiated Thyroid Carcinoma; **TME**:Tumor Mutation Burden; **UMAP**: Uniform Manifold Approximation and Projection; **GSVA**: Gene Set Variation Analysis; **WES**: Whole‐Exome Sequencing; **inferCNV**: Inferred Copy Number Variation. The violin plots in panel H were analyzed using one‐way ANOVA followed by Bonferroni correction for multiple comparisons, while the box plots were evaluated using paired t‐tests. ^*^ *p* < 0.05, ^**^ *p* < 0.01, ^***^ *p* < 0.001, ^****^ *p* < 0.0001.

Using the Harmony algorithm, we integrated multi‐sample spRNAseq data and identified 16 niches. Based on histopathologic features and marker genes, niches were annotated as four DTC niches (CITED1⁺, S100A1⁺, DNAJA4⁺, NPW⁺), three HGFCTC niches (SIGLEC6⁺, SLC34A2⁺, BMP8A⁺), two ATC niches (SERPINE1⁺, KRT5⁺), three immune‐enriched niches (CCL9⁺, FOSB⁺, MARCO⁺), three fibroblast‐related niches (SFRP4⁺ fibroblast, DES⁺ myocyte, TTN⁺ myocyte), and one NSE niche (KRT4⁺) (**Figure** **2**A–C; Figure S5A,B and Table S3, Supporting Information). Notably, correlation analysis between pathological annotation and niche annotation, along with UMAP visualization, revealed a distinct transcriptomic boundary between ATC and non‐ATC regions, whereas no clear demarcation was observed between DTC and HGFCTC subtypes (Figure 2B; Figure S5C,D, Supporting Information). Thyroid differentiation‐related genes were predominantly expressed in DTC niches, particularly CITED1⁺ DTC, and gradually decreased toward ATC niches (Figure 2D). Consistent with histologic classification, ATC regions could be subdivided into SERPINE1⁺ ATC, enriched in spindle cells, and KRT5⁺ ATC, characterized by squamous morphology.^[^ ^19^ ^]^

Cellular proportion analysis revealed significant inter‐sample variability among DTC niches. For example, NPW⁺ DTC was detected only in the P32 sample (Figure 2E,F; Figure S5E, Supporting Information), whereas CITED1⁺ DTC, SIGLEC6⁺ HGFCTC, and SERPINE1⁺ ATC were present across multiple samples. Pathway analysis showed that tumor‐associated malignant signaling pathways were more prominently activated in ATC niches compared to DTC and HGFCTC niches (Figure 2G; Figure S5F,G, Supporting Information). To further dissect intratumoral heterogeneity, non‐negative matrix factorization (NMF) clustering was performed on tumor regions, identifying nine transcriptional modules whose spatial distribution was largely consistent with histopathological annotations (Figure S6A–C, Supporting Information). Inference of Copy Number Variations (InferCNV) analysis using normal thyroid epithelial cells as a reference showed that, overall, there was no statistically significant difference in CNV levels between ATC regions and co‐existing DTC regions, although sample‐specific heterogeneity was observed (Figure 2H; Figure S6D,E, Supporting Information). Further wholeexome sequencing (WES) analysis of regions with different differentiation states within the same specimen revealed that co‐existing DTC regions exhibited a tumor mutation burden (TMB) comparable to that of adjacent ATC regions (Figure 2I; Figure S6F, Supporting Information). Notably, several key dedifferentiation‐related mutations, such as in ATR and BRCA2, were already present in the DTC regions.

Moreover, collectively, these findings indicate that co‐existing DTC regions have already acquired genomic features resembling those of ATC. However, at the transcriptomic level, they still exhibit substantial heterogeneity during the transition toward dedifferentiation, suggesting the presence of diverse regulatory mechanisms and evolutionary trajectories in this intermediate state.

### 2.3 Pseudotime Analysis Uncovers Transcriptomic Changes During DTC‐to‐ATC Transition

**Figure 3:**
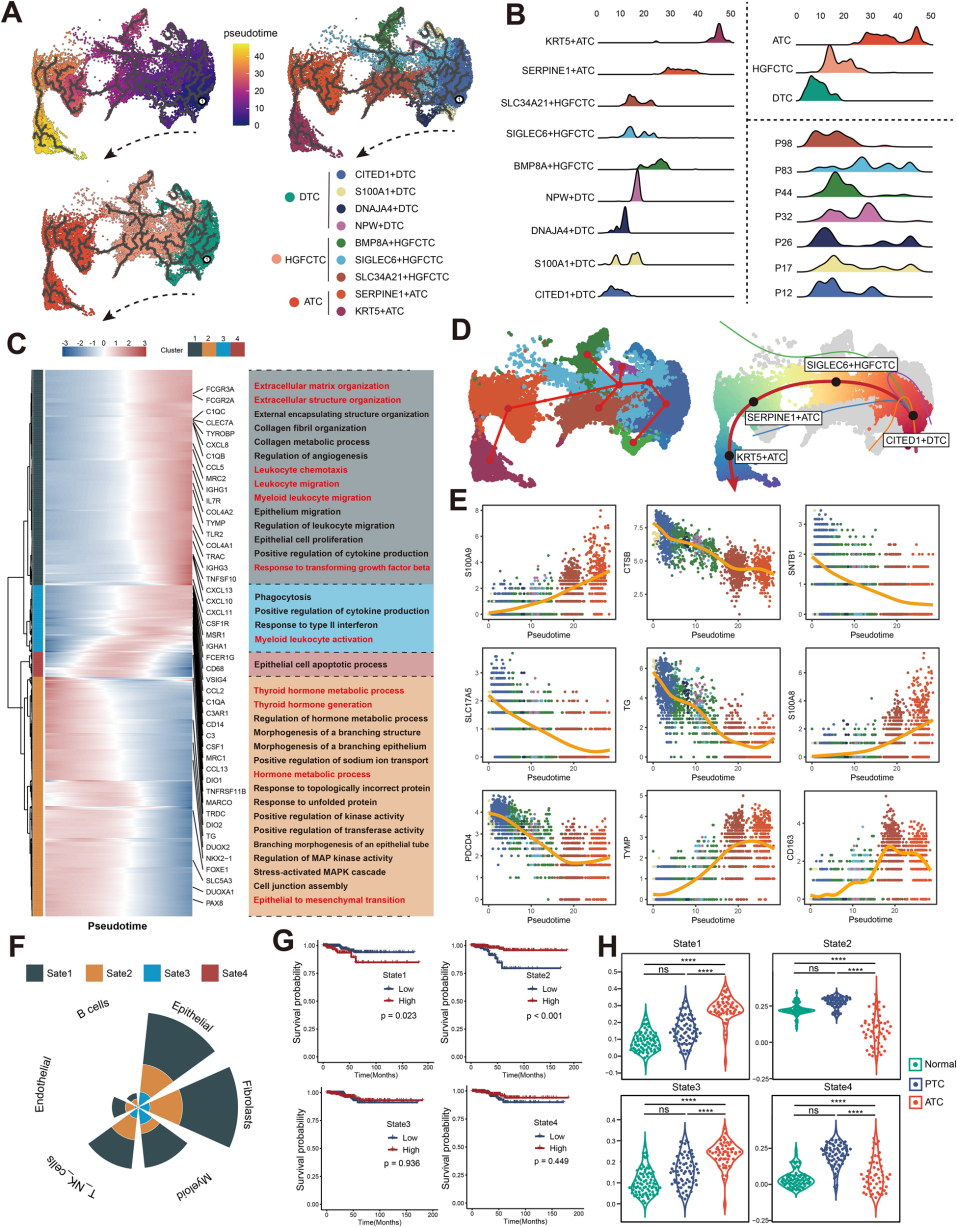
Pseudotime analysis of spRNAseq in ATC/PDTC‐DTC coexisting samples. A) Pseudotime trajectory of DTC‐to‐ATC differentiation inferred by Monocle3. B) Distribution of pseudotime across different tumor niches, histopathological regions, and samples. C) Heatmap showing gene modules clustered based on pseudotime‐associated expression dynamics inferred by monocle3. D) DTC‐to‐ATC differentiation trajectories reconstructed using the Slingshot algorithm. E) Key genes dynamically altered along the pseudotime trajectory as determined by Slingshot. F) Distribution of pseudotime‐specific gene modules across single‐cell subpopulations. G) Prognostic relevance of distinct pseudotime states based on TCGA cohort analysis. H) Expression scores of pseudotime states across normal thyroid tissue, PTC, and ATC samples in the GEO dataset (GSE33630). **ATC**: Anaplastic Thyroid Carcinoma; **DTC**: Differentiated Thyroid Carcinoma; **PDTC**: Poorly Differentiated Thyroid Carcinoma; **HGFCTC**: High‐Grade Follicular Cell‐derived Thyroid Carcinoma; **spRNA‐seq**: Spatial Transcriptomic RNA Sequencing; **PTC**: Papillary Thyroid Carcinoma; **TCGA**: The Cancer Genome Atlas; **GEO**: Gene Expression Omnibus. Significance in G was analyzed using the log‐rank test for survival analysis; Significance in H was determined using one‐way ANOVA and Bonferroni multiple comparison test ^*^ *p* < 0.05, ^**^ *p* < 0.01, ^***^ *p* < 0.001, ^****^ *p* < 0.0001.

Using Monocle3, we reconstructed the pseudotime trajectory underlying the transition from DTC to ATC, capturing dynamic transcriptomic changes during dedifferentiation (**Figure** **3**A). This trajectory mirrored the stepwise progression from CITED1⁺ DTC to KRT5⁺ ATC, a squamoid subtype marking the terminal state (Figure 3B). Pseudotime‐associated genes were clustered into four transcriptional states: states 1 and 3 included genes upregulated along dedifferentiation, state 2 comprised downregulated genes, and state 4 displayed transient activation (Figure 3C; Table S4, Supporting Information). Functional enrichment revealed that state 1 genes were associated with extracellular matrix remodeling and macrophage infiltration, while state 4 genes were linked to thyroid hormone metabolism and epithelial transdifferentiation. State scores within individual samples closely tracked the pseudotime dedifferentiation trajectory, supporting their potential as markers for assessing thyroid cancer dedifferentiation (Figure S7, Supporting Information).

Furthermore, Slingshot pseudotime analysis validated the dedifferentiation trajectory inferred by Monocle3 and delineated a continuous path from CITED1⁺ DTC through SIGLEC6⁺ HGFCTC and SERPINE1⁺ ATC to KRT5⁺ ATC (Figure 3D). Other niches may represent branching subtypes that emerge during this dedifferentiation process (Figure 3D). We also identified nine core regulators—*S100A9*, *CTSB*, *SNTB1*, *SLC17A5*, *TG*, *S100A8*, *PDCD4*, *TYMP*, and *CD163*—that were tightly co‐expressed with the dedifferentiation trajectory (Figure 3E). To account for spRNAseq heterogeneity, we integrated multiple scRNAseq datasets and found these trajectory‐related states were enriched in epithelial cells, fibroblasts, myeloid cells, and T cells (Figure 3F; Figure S8A,B, Supporting Information). Clinically, high expression of state 1 and low expression of state 2 were significantly associated with poor prognosis in The Cancer Genome Atlas (TCGA) cohort (Figure 3G), and the gradient of state scores across histological subtypes was validated in an independent Gene Expression Omnibus (GEO) dataset (Figure 3H).

Collectively, our analyses define four transcriptional states driving thyroid cancer dedifferentiation, closely linked to TIME remodeling. These findings highlight a coordinated reprogramming of tumor‐intrinsic and immunosuppressive features during progression.

### 2.4 TYMP⁺ TAMs Progressively Accumulate During Thyroid Cancer Dedifferentiation

**Figure 4:**
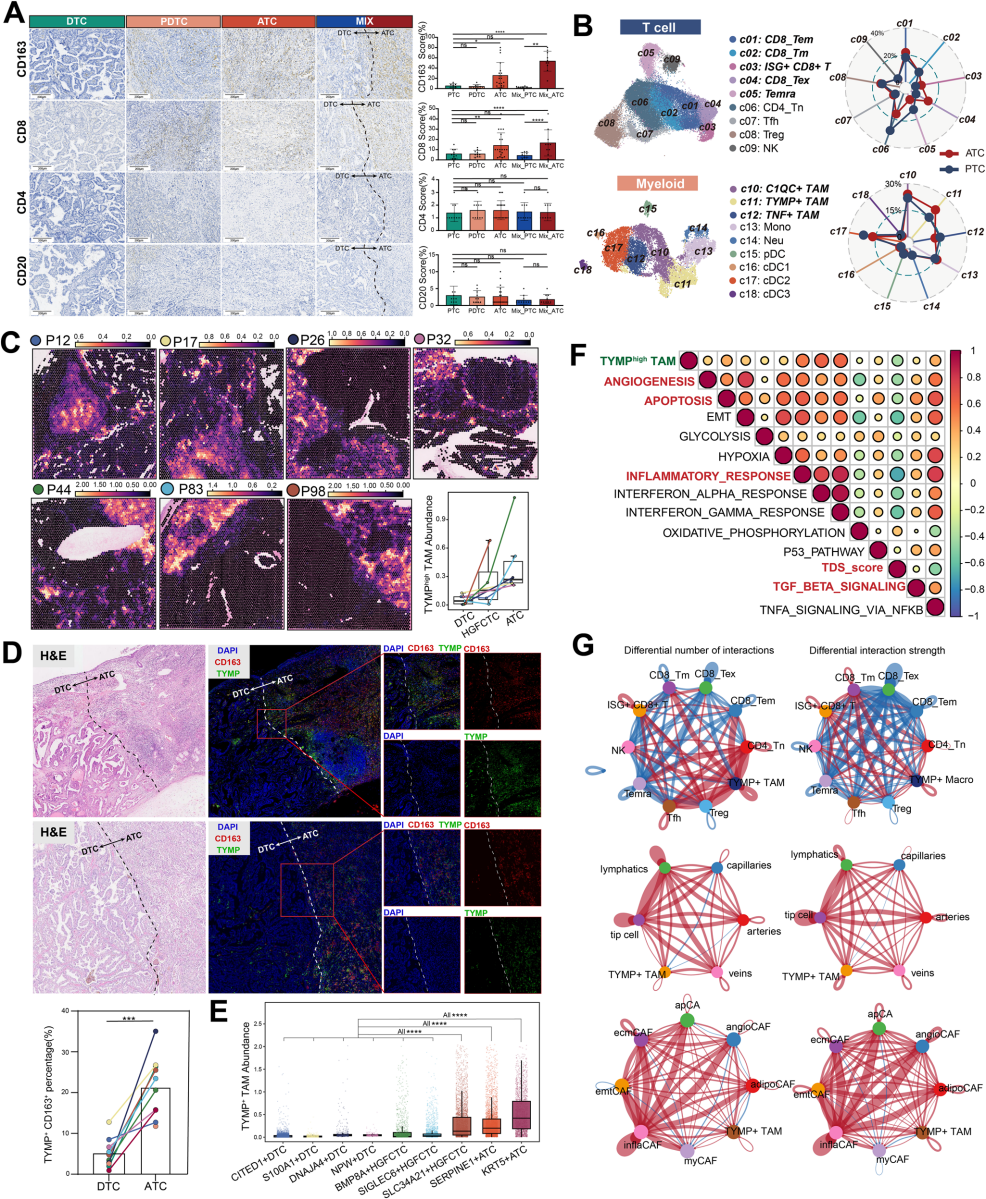
SpRNAseq analysis of immune infiltration in ATC/PDTC‐DTC coexisting samples. A) Expression patterns of CD163, CD8, CD4, and CD20 across different histological subtypes of thyroid carcinoma identified by IHC. B) Single‐cell subcluster analysis of T cells and myeloid cells using public databases. C) Integrated analysis of spRNA‐seq and scRNA‐seq using the Cell2location algorithm revealed the spatial distribution of TYMP⁺ TAMs in ATC/PDTC‐DTC coexisting samples. D) MIF validation of TYMP⁺ TAMs in ATC‐DTC coexisting samples. E) Distribution of TYMP⁺ TAMs across different tumor niches in spRNAseq. F) Correlation between TYMP⁺ TAMs and pathway scores related to tumor and immune responses in spRNAseq analysis. G) Cell‐cell communication analysis of TYMP⁺ TAMs with T cells, endothelial cells, and fibroblast subpopulations based on scRNAseq data. **ATC**: Anaplastic Thyroid Carcinoma; **DTC**: Differentiated Thyroid Carcinoma; **HGFCTC**: High‐Grade Follicular Cell‐derived Thyroid Carcinoma; **IHC**: Immunohistochemistry; **PDTC**: Poorly Differentiated Thyroid Carcinoma; **spRNA‐seq**: Spatial RNA Sequencing; **scRNA‐seq**: Single‐Cell RNA Sequencing; **TAM**: Tumor‐Associated Macrophage; **TYMP**: Thymidine Phosphorylase; **MIF**: Multiplex Immunofluorescence. Significance in D was analyzed using the log‐rank test for survival analysis; Significance in A, E was determined using one‐way ANOVA and Bonferroni multiple comparison test ^*^ *p* < 0.05, ^**^ *p* < 0.01, ^***^ *p* < 0.001, ^****^ *p* < 0.0001.

To elucidate immune microenvironmental alterations during dedifferentiation, immunohistochemistry (IHC) revealed increased infiltration of CD163^+^ myeloid cells and CD8^+^ T cells in poorly differentiated regions, while CD4^+^ and CD20^+^ cells remained sparse and unchanged (**Figure** **4**A). Publicly scRNAseq identified distinct subsets within CD8^+^ T cells and myeloid populations, including CD8_Tem, CD8_Tm, ISG^+^ CD8 T, Temra, C1QC^+^ TAMs, TYMP^+^ TAMs, and TNF^+^ TAMs (Figure 4B; Figure S8C–H, Supporting Information). Thymidine phosphorylase (TYMP) plays a key role in promoting tumor angiogenesis, invasion, and metastasis, as well as in modulating the TIME.^[^ ^21^ ^]^ It also serves as a core gene involved in the previously mentioned dedifferentiation process (Figures 1E and 3E). Spatial transcriptomic deconvolution via cell2location demonstrated selective enrichment of TYMP^+^TAMs in dedifferentiated regions, with markedly reduced infiltration in well‐differentiated areas (Figure 4C; Figure S10, Supporting Information). This distribution was corroborated by multiplex immunofluorescence (MIF), which showed robust accumulation of TYMP^+^CD163^+^ macrophages in ATC regions (Figure 4D). TYMP⁺ TAMs were predominantly enriched within the niche of SLC34A21⁺ HGFCTC, SEPRINE1^+,^ and KRT5^+^ ATC (Figure 4E).

Spatial pathway enrichment analysis revealed strong associations between TYMP^+^ TAMs infiltration and angiogenesis, apoptosis, inflammatory response, and TGF‐β signaling (Figure 4F). Furthermore, cell–cell communication analysis showed enhanced interactions between TYMP^+^ TAMs and CD8^+^ T cells, endothelial cells, and fibroblasts, indicating a potential role in reshaping the immune landscape and promoting tumor progression (Figure 4G; Figure S9A–F, Supporting Information). Collectively, these findings suggest that M2‐like myeloid cells, particularly TYMP^+^ TAMs, undergo spatial and functional reprogramming during dedifferentiation and may serve as critical mediators of the aggressive phenotype in thyroid cancer.

### 2.5 PDCD4 Loss Promotes M2 Polarization of Macrophages in Thyroid Cancer

**Figure 5:**
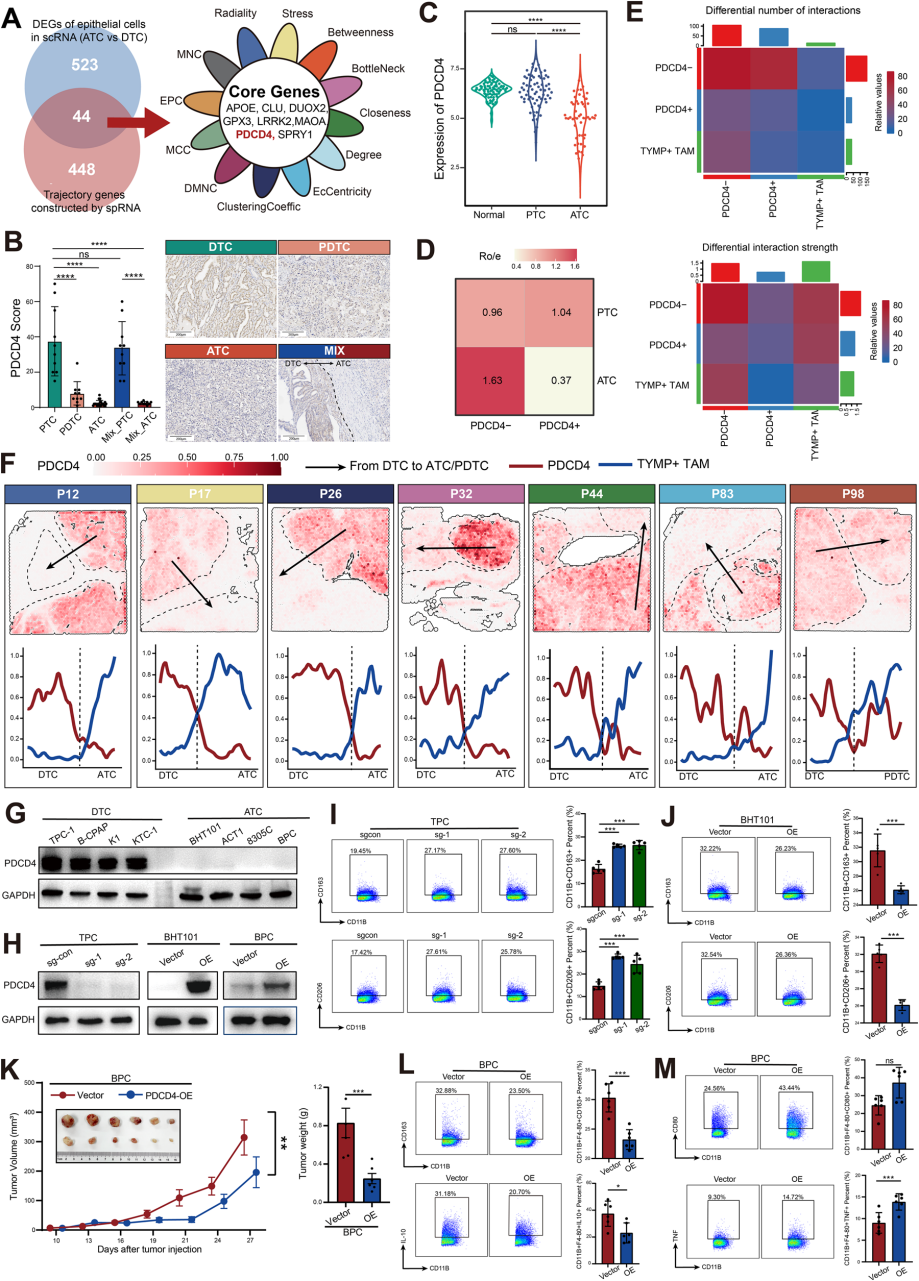
Loss of PDCD4 expression is associated with M2 macrophage enrichment in ATC. A) Hub gene analysis of dedifferentiation‐associated genes in ATC tumor cells based on integrated spRNAseq and scRNAseq data. B) IHC of PDCD4 across different histological subtypes of thyroid carcinoma. C) Expression levels of PDCD4 in normal thyroid tissue, PTC, and ATC in the GEO dataset GSE33630. D) Proportion of PDCD4‐positive and ‐negative tumor cell populations in ATC and PTC based on scRNAseq. E) Cell‐cell communication analysis between PDCD4‐positive/negative tumor cells and TYMP⁺ TAMs. F) Spatial localization of PDCD4 and TYMP⁺ TAMs in spRNA‐seq. G) WB analysis of PDCD4 expression in thyroid cancer cell lines representing different histological subtypes. H) WB validation of PDCD4 knockout in the DTC cell line (TPC‐1) and PDCD4 overexpression in ATC cell lines (BHT101 and BPC). I,J) FCM analysis showing that PDCD4 knockout in TPC‐1 promotes M2 polarization of THP‐1‐derived macrophages (I), while PDCD4 overexpression in BHT101 inhibits M2 polarization (J). K) Tumor growth curve and tumor weight boxplot showing that PDCD4 overexpression in the murine ATC cell line BPC suppresses tumor progression in vivo. L–M) FCM showing that PDCD4 overexpression in BPC reduces M2 macrophage (L) infiltration and promotes M1 macrophage (M) infiltration in tumors. **ATC**: Anaplastic Thyroid Carcinoma; **DTC**: Differentiated Thyroid Carcinoma; **FCM**: Flow cytometric; **IHC**: Immunohistochemistry; **PTC**: Papillary Thyroid Carcinoma; **PDCD4**: Programmed Cell Death 4; **TAM**: Tumor‐Associated Macrophage; **TYMP**: Thymidine Phosphorylase; **scRNA‐seq**: Single‐Cell RNA Sequencing; **spRNA‐seq**: Spatial Transcriptomic RNA Sequencing; **GEO**: Gene Expression Omnibus; **WB**: Western Blot. Significance in B, C, I, and K was determined using one‐way ANOVA and the Bonferroni multiple comparison test. Significance in J, L, M was determined using Unpaired t‐test ^*^ *p* < 0.05, ^**^ *p* < 0.01, ^***^ *p* < 0.001, ^****^ *p* < 0.0001.

Programmed cell death 4 (PDCD4) was identified as a core gene in the state 2 phase during the dedifferentiation trajectory from DTC to ATC, and scRNAseq analysis revealed it as a key DEG between malignant epithelial cells of ATC and DTC (**Figure** **5**A; Figure S12, Supporting Information). IHC demonstrated a progressive loss of PDCD4 expression with decreasing tumor differentiation (Figure 5B), consistent with its significant downregulation in ATC tissues based on GEO datasets (Figure 5C). Stratifying malignant epithelial cells in scRNAseq dataset based on the median PDCD4 expression, we observed significantly enhanced cell–cell interactions between PDCD4− cells and TYMP⁺ TAMs (Figure 5D,E; Figure S9G,H, Supporting Information). SpRNAseq further revealed that PDCD4‐low regions were often enriched with TYMP⁺ TAMs (Figure 5F).

Western blotting (WB) analysis across thyroid cancer cell lines showed that PDCD4 expression was markedly reduced or absent in ATC cell lines (BHT101, ACT1, 8305C, BPC (mouse‐derived) compared to DTC cell lines (TPC‐1, B‐CPAP, K1, KTC‐1) (Figure 5G; Figure S11A, Supporting Information). We then generated PDCD4 knockout TPC‐1 cells and PDCD4‐overexpressing BHT101 and BPC cells (Figure 5H; Figure S11B, Supporting Information). The conditioned media from these cells were used in M2 macrophage differentiation assays with THP‐1 and RAW 264.7 cells to evaluate whether supernatants from different groups (PDCD4 knockout or overexpression) could enhance the M2 macrophage phenotype (Figure S13A, Supporting Information). Flow cytometry (FCM) revealed that PDCD4 knockout in TPC‐1 significantly increased the proportion of CD11B⁺CD163⁺ and CD11B⁺CD206⁺ cells, whereas PDCD4 overexpression in BHT101 and BPC yielded the opposite effect (Figure 5I,J; Figure S13B–D, Supporting Information). In vivo, PDCD4 overexpression in BPC cells led to a significant reduction in subcutaneous tumor volume (Figure 5K). FCM analysis of the tumors showed a marked decrease in CD11B⁺CD163⁺ and CD11B⁺IL10⁺ cells, alongside an increase in CD11B⁺CD80⁺, and CD11B⁺TNF⁺ cells (Figure 5L,M; Figure S13C, Supporting Information).

Collectively, these data indicate that sustained downregulation of PDCD4 during thyroid cancer dedifferentiation is associated with enhanced M2‐like macrophage infiltration and immunosuppressive remodeling, potentially driving tumor progression.

### 2.6 PDCD4 Deficiency Enhances Translation of M2‐Related Proteins via an eIF4A‐Dependent Mechanism

**Figure 6:**
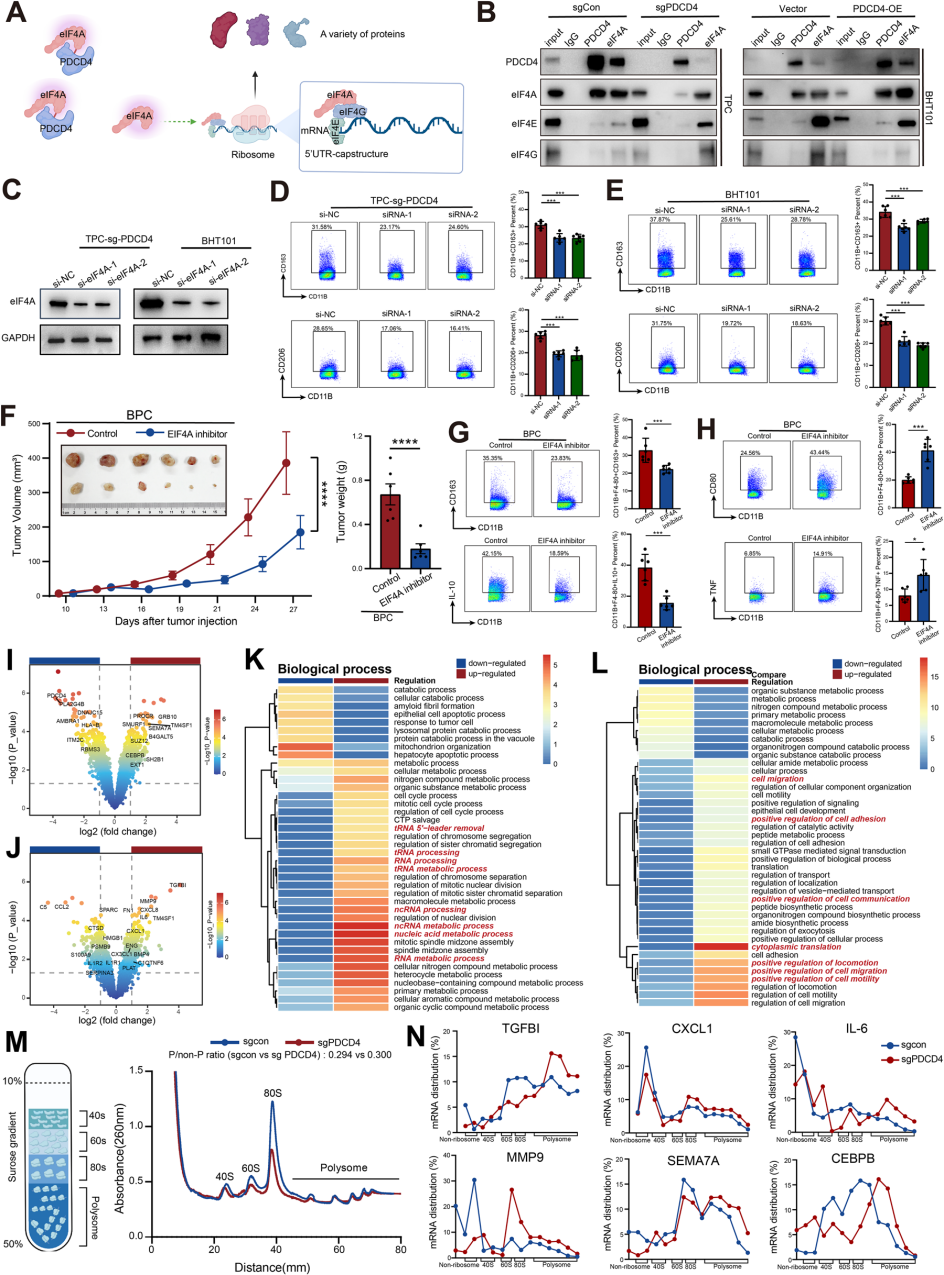
PDCD4 regulates the secretion of M2‐polarizing proteins in an eIF4A‐dependent manner. A) Schematic diagram of PDCD4–eIF4A‐mediated regulation of protein expression patterns. B) Co‐IP analysis showing the changes in the eIF4F complex (eIF4A–eIF4E–eIF4G) upon PDCD4 knockout or overexpression in BHT101 cells. C) WB analysis confirmed the knockdown efficiency of eIF4A using siRNA in TPC‐sg‐PDCD4 and BHT101. D,E) FCM analysis showing that inhibition of eIF4A reduced M2 macrophage polarization induced by TPC‐sg‐PDCD4 (D) and BHT101 (E) cells in THP‐1 polarization assays. F) In vivo experiments showed that the eIF4A inhibitor suppressed subcutaneous tumor growth in mice. G,H) FCM revealed that eIF4A inhibition decreased M2 macrophage infiltration (G) and increased M1 macrophage infiltration (H) in tumors. I–L) Proteomic and pathway enrichment analyses of intracellular proteins (I, K) and secreted proteins in the supernatant (J, L) after PDCD4 knockout in TPC‐1 cells. M,N) Polysome profiling analysis demonstrated that PDCD4 knockout affected the translational efficiency of specific mRNAs in TPC‐1 cells. **Co‐IP**: Co‐immunoprecipitation**; eIF4A**: eukaryotic Initiation Factor 4A**; eIF4E**: eukaryotic Initiation Factor 4E**; eIF4G**: eukaryotic Initiation Factor 4G**; FCM**: Flow cytometry; **PDCD4**: Programmed Cell Death 4; **WB**: Western Blot. Significance in D‐F was determined using one‐way ANOVA and the Bonferroni multiple comparison test. Significance in G, H was determined using Unpaired t‐test ^*^ *p* < 0.05, ^**^ *p* < 0.01, ^***^ *p* < 0.001, ^****^ *p* < 0.0001.

Previous studies have shown that PDCD4 suppresses translation by binding to eukaryotic initiation factor 4A (eIF4A) and inhibiting the assembly of the translation initiation complex.^[^ ^22^, ^23^ ^]^ Loss of PDCD4 leads to aberrant activation of eIF4A and increased global protein translation (**Figure** **6**A). WB analysis showed that PDCD4 knockout or overexpression did not alter eIF4A protein levels (Figure S11C, Supporting Information). However, co‐immunoprecipitation (Co‐IP) assays revealed that PDCD4 knockout enhanced the formation of the eIF4A–eIF4E–eIF4G complex, whereas PDCD4 overexpression in BHT101 cells reduced its assembly (Figure 6B; Figure S11D, Supporting Information).To investigate the functional consequence of this pathway, eIF4A was silenced in TPC‐sg‐PDCD4 and BHT101 cells using siRNA (Figure 6C; Figure S11E, Supporting Information). Subsequent THP‐1 co‐culture assays revealed that eIF4A knockdown significantly reduced the generation of CD11B⁺CD163⁺ and CD11B⁺CD206⁺ macrophages (Figure 6D,E). Similarly, in RAW264.7 polarization assays, the conditioned medium from eIF4A‐knockdown BPC cells yielded consistent results (Figure S13E,F, Supporting Information). In vivo, subcutaneous implantation of BPC cells followed by eIF4A inhibitor treatment resulted in marked tumor growth suppression (Figure 6F). FCM analysis of tumors showed decreased infiltration of M2‐like macrophages (CD11B⁺CD163⁺ and CD11B⁺IL10⁺) and a concurrent increase in M1‐like macrophages (CD11B⁺CD80⁺ and CD11B⁺TNF⁺) after eIF4A inhibitor treatment (Figure 6G,H).

To further explore the translational landscape under PDCD4 deficiency, we performed proteomic profiling of PDCD4‐knockout TPC‐1 cells and their conditioned media. Loss of PDCD4 upregulated intracellular proteins such as CEBPB, SEMA7A, GRB10, and TM4SF1, along with secreted factors including TGFBI, MMP9, CXCL8, IL6, and CXCL1—molecules implicated in M2 polarization, extracellular matrix remodeling, and immunosuppressive niche formation (Figure 6I,J; Tables S5 and S6, Supporting Information). Gene Ontology (GO) enrichment analysis demonstrated upregulation of translation‐related pathways intracellularly, while secreted proteins were enriched for cell communication and migration signatures (Figure 6K,L). Polysome profiling further confirmed enhanced translational efficiency in PDCD4‐deficient cells, particularly for key immunomodulatory proteins such as *TGFBI*,^[^ ^24^ ^]^ *CXCL1*,^[^ ^25^ ^]^ *IL‐6*,^[^ ^26^ ^]^ *MMP9*,^[^ ^27^ ^]^ *SEMA7A*,^[^ ^28^ ^]^ and *CEBPB* ^[^ ^29^ ^]^ (Figure 6M,N; Table S7, Supporting Information).

Collectively, these findings suggest that PDCD4 loss reprograms the tumor secretome and promotes an immunosuppressive microenvironment through eIF4A ‐dependent translational activation.

## 3 Discussion

ATC is one of the most aggressive thyroid malignancies, arising from the dedifferentiation of DTC, and remains largely resistant to current treatment modalities.^[^ ^1^, ^2^, ^3^ ^]^ Compared with DTC, ATC displays a profoundly immunosuppressive tumor microenvironment, characterized by enriched M2‐like macrophages and cancer‐associated fibroblasts.^[^ ^7^, ^16^, ^17^ ^]^ Dissecting the dynamic changes in gene expression and immune landscape during the dedifferentiation process may offer novel therapeutic insights for ATC. In this study, we applied spRNAseq to paired tumor specimens containing both ATC and co‐existing DTC regions, enabling the reconstruction of a pseudotemporal trajectory that delineates the transition from DTC to ATC. Mechanistically, we identified a marked loss of PDCD4 expression in ATC regions, which led to aberrant activation of eIF4A‐dependent M2‐polarizing factors and ultimately contributed to the establishment of an immunosuppressive microenvironment in ATC.

The progression from DTC to ATC involves a multilayered dedifferentiation process spanning the genomic, transcriptomic, proteomic, and TIME levels.^[^ ^30^ ^]^ Genetically, this transition is often initiated by the stepwise accumulation of additional oncogenic events—such as TP53 and TERT promoter mutations—on top of pre‐existing drivers like BRAFV600E or RAS mutations.^[^ ^9^, ^10^ ^]^ Consistent with previous studies, our findings revealed that the co‐existing DTC regions, although lacking the overt pathological features of ATC, had already acquired a high mutational burden and harbored several key genetic alterations commonly observed in ATC.^[^ ^10^ ^]^ At the transcriptomic level, widespread reprogramming occurs, including silencing of thyroid differentiation markers and activation of gene networks associated with epithelial–mesenchymal transition, stemness, and proliferation.^[^ ^31^ ^]^ Concurrently, enhanced activity of translation initiation factors such as eIF4A promotes selective translation of mRNAs involved in immune evasion and tumor invasion. These changes converge at the proteomic level to reinforce malignant phenotypes. In parallel, the TIME undergoes extensive remodeling, characterized by diminished immune surveillance and substantial enrichment of M2‐like TAMs and activated cancer‐associated fibroblasts, which together foster a profoundly immunosuppressive and tumor‐permissive niche.

The transition from DTC to ATC is not a single linear event, but rather a complex, multi‐stage evolutionary process that gives rise to a spectrum of pathological subtypes. Among these, PDTC and the more recently recognized HGDTC represent key intermediate states.^[^ ^19^ ^]^ PDTC typically exhibits partial loss of differentiation and architectural disarray, whereas HGDTC retains differentiated structures but displays marked nuclear atypia.^[^ ^32^, ^33^, ^34^ ^]^ Our data reveal that, despite their distinct histopathological appearances, PDTC and HGDTC share highly similar transcriptomic features. This suggests that they may represent a continuum of states within the dedifferentiation process, and together constitute a transcriptionally defined intermediate stage toward ATC (SIGLEC6+HGFCTC). Moreover, the dedifferentiation of DTC can give rise to a wide range of aggressive pathological features, reflecting significant phenotypic heterogeneity (NPW+DTC etc.).^[^ ^35^ ^]^ This may account for the development of aggressive variants of papillary thyroid carcinoma, including the tall cell variant and columnar cell variant, both of which exhibit molecular profiles resembling those seen in dedifferentiated states. Notably, two distinct histological patterns—spindle cell and squamous cell morphologies—are frequently observed within ATC.^[^ ^36^ ^]^ Our transcriptomic analysis indicates that while these two forms share common features in immune microenvironment composition, tumorigenic signaling, and inflammatory pathway activity, they also possess unique molecular signatures.

Despite the marked histologic diversity and intratumoral heterogeneity observed during the progression from DTC to ATC, a consistent immunologic feature is the sustained accumulation of M2‐polarized TAMs within the TIME. This enrichment suggests that M2 macrophage–mediated immunosuppression may represent a critical barrier—and therapeutic target—during tumor dedifferentiation.^[^ ^7^, ^37^ ^]^ PD‐1/PD‐L1 immune checkpoint inhibitors (ICIs) have become first‐line therapies for various solid tumors.^[^ ^38^, ^39^, ^40^ ^]^ Although ICIs have shown some clinical benefits in ATC, overall response rates remain limited, indicating persistent immunosuppressive mechanisms that ICIs alone fail to overcome.^[^ ^41^ ^]^ While ATC exhibits greater CD8⁺ T cell infiltration than DTC, the TIME remains profoundly immunosuppressive, largely driven by M2 TAMs. These cells not only suppress T cell function through direct interactions but also secrete immunosuppressive and pro‐angiogenic mediators, including TYMP and TGFB, which further reinforce immune evasion and tumor progression.^[^ ^42^, ^43^, ^44^ ^]^ Given this context, ICIs monotherapy may be insufficient to mount effective antitumor immunity. Combination strategies targeting M2 macrophages and reprogramming the TIME may be essential to enhance therapeutic efficacy and improve immunotherapy responses in ATC.^[^ ^45^ ^]^

Our study demonstrates that targeting the PDCD4–eIF4A axis represents a novel strategy to reprogram the TIME in ATC. PDCD4, a well‐established tumor suppressor and translational repressor, has been implicated in multiple aspects of tumor biology, including initiation, progression, metastasis, and therapeutic responsiveness.^[^ ^22^ ^]^ Mechanistically, PDCD4 inhibits the activity of eIF4A, thereby disrupting the cap‐dependent translation of a subset of oncogenic and immunosuppressive mRNAs.^[^ ^23^, ^46^ ^]^ Notably, a study by Brina et al. reported that PDCD4 expression is suppressed in the absence of functional p53, which may partially explain the marked downregulation of PDCD4 observed in dedifferentiated thyroid carcinomas such as ATC.^[^ ^47^ ^]^ The loss of PDCD4 leads to aberrant activation of eIF4A, contributing to an immunosuppressive and tumor‐promoting microenvironment.^[^ ^47^, ^48^, ^49^ ^]^ Pharmacologic inhibition of eIF4A has been shown to effectively block M2 macrophage polarization, reduce the secretion of immunosuppressive cytokines, and drug resistance, especially in BRAFV600‐mutant tumours.^[^ ^50^, ^51^, ^52^ ^]^ Thus, restoring PDCD4 expression or directly inhibiting eIF4A may facilitate a phenotypic shift of TAMs toward a proinflammatory M1‐like state, suppress tumor angiogenesis, and ultimately improve responsiveness to ICIs. These findings underscore the therapeutic potential of targeting translational control pathways to overcome immune resistance in ATC.

eIF4A, a key regulator of translation initiation, is an emerging target in cancer therapy. Zotatifin, the first‐in‐class eIF4A inhibitor, has entered phase I/II trials in solid tumors such as triple‐negative breast and prostate cancer (e.g., NCT04092673), showing early signs of efficacy and good tolerability.^[^ ^51^, ^53^, ^54^ ^]^ However, clinical translation faces challenges. As eIF4A is essential for normal cells, its inhibition may cause dose‐dependent toxicity, including myelosuppression and gastrointestinal side effects.^[^ ^53^, ^54^ ^]^ Most inhibitors require injection, and tumor‐specific delivery systems or oral formulations are lacking. Moreover, tumor sensitivity varies, emphasizing the need for predictive biomarkers. Our study highlights the potential of eIF4A inhibition in reshaping the immune microenvironment in ATC, supporting its therapeutic relevance. Overall, while promising, the broader application of eIF4A inhibitors requires optimization of safety, delivery, and patient selection.

This study provides new mechanistic insights into the dedifferentiation process of thyroid cancer and offers potential directions for optimizing its immunotherapeutic strategies. However, several important questions remain to be addressed. First, a comprehensive dedifferentiation atlas could be constructed by integrating multi‐omics data, including proteomics, and incorporating solitary DTC samples as controls to better understand the origin of dedifferentiation. Second, the upstream signals regulating PDCD4 expression—such as p53 loss—and its potential cooperation with other translational regulators warrant further investigation. Third, the lack of basic research tools (e.g., murine DTC cell lines) limits systematic exploration of thyroid cancer dedifferentiation. Finally, the rarity of ATC results may reduce the statistical power and limit the generalizability of the findings. In summary, further mechanistic and translational studies are needed to validate the proposed pathways and therapeutic potential in ATC.

## 4 Conclusion

Our study delineates a multistep dedifferentiation axis in thyroid carcinogenesis, wherein coexisting DTC regions already harbor ATC‐like genomic features. Through spRNAseq, we reconstructed a dedifferentiation trajectory (CITED1⁺ DTC → SIGLEC6⁺ HGFCTC → SERPINE1⁺ ATC → KRT5⁺ ATC) and identified PDCD4 as a central regulator driving transcriptional reprogramming across four distinct states. Notably, ATC regions exhibited significant enrichment of TYMP⁺ TAMs, establishing an immunosuppressive microenvironment. Mechanistically, PDCD4 suppresses the production of tumor‐derived M2‐polarizing factors by binding to eIF4A, whereas its loss induces eIF4A‐dependent overtranslation of immunosuppressive proteins. These findings not only uncover PDCD4‐eIF4A axis dysfunction as a key driver of TAMs‐mediated immune evasion in ATC but also propose targeting eIF4A as a novel therapeutic strategy to reprogram the immunosuppressive niche.

## 5 Experimental Section

### Patient and Sample Selection

From 23239 thyroid cancer patients treated at Sun Yat‐sen University Cancer Center (SYSUCC) between 2010–2024, 21111 non‐ATC/HGFCTC cases were excluded, 128 metastases from other sites, and 1921 specimens with unavailable tissues. Among 79 surgically resected ATC/HGFCTC cases, 47 lacking DTC components and 19 with <10% mixed regions were excluded. After quality control, eliminating 4 substandard samples and 2 with section detachment, 7 ATC/HGFCTC mixed specimens were ultimately included for spRNAseq (Figures S1 and S2, Supporting Information). To minimize diagnostic bias, all histopathological assessments were conducted independently by two board‐certified pathologists blinded to clinical outcomes. Initial specimen eligibility determination (ATC/HGFCTC with ≥10% DTC components) required consensus between both evaluators. In addition, supplementary cohorts of DTC, PDTC, and ATC were compiled for specimen validation. Comprehensive clinical data, including baseline characteristics, treatment regimens, and outcomes, were collected for all patients included in this study.

This study protocol was approved by the Medical Ethics Committee of SYSUCC (B2025‐133‐01). All procedures were conducted in accordance with the ethical principles outlined in the Declaration of Helsinki. Given the retrospective design of the study and the use of archived clinical specimens and records, the Ethics Committee granted a waiver of written informed consent.

### SpRNA‐seq and scRNA‐seq Data Processing

SpRNA was employed to analyze the spatial distribution of gene expression in formalin‐fixed, paraffin‐embedded (FFPE) thyroid carcinoma tissue samples. FFPE tissue sections (10 µm thick) were mounted onto Visium spatial transcriptomics slides (10x Genomics) and subsequently subjected to deparaffinization, antigen retrieval, and HE staining to preserve tissue morphology. Following staining, RNA molecules were photoreleased and captured by spatial barcode probes on the slide. RNA was then reverse‐transcribed, and sequencing libraries were constructed using the Visium FFPE reagent kit, strictly adhering to the manufacturer's protocol. The resulting libraries were sequenced on the Illumina NovaSeq platform to generate high‐resolution spatial transcriptomic data.

ScRNA‐seq data for ATC and DTC were obtained from publicly available datasets, including GSE148673, GSE184362, GSE193581, and GSE210347.^[^ ^7^, ^55^, ^56^, ^57^ ^]^ A total of 37 samples were analyzed, comprising 19 DTC and 18 ATC samples. All scRNAseq data underwent standardized quality control and analysis to ensure consistency and reliability.

### Dimensionality Reduction and Clustering Analysis

After quality control of spRNAseq data, the dataset was processed using the Space Ranger pipeline (10x Genomics), including alignment, barcode assignment, and gene expression quantification. Normalization was performed using the “SCTransform” function in the Seurat package (R) to correct for technical variability. Principal component analysis (PCA) was conducted with “RunPCA”, followed by spatial clustering using the shared nearest neighbor (SNN) algorithm via “FindClusters”. The spatial distribution of different clusters was visualized using “SpatialDimPlot”. Marker genes for each spatial cluster were identified using “FindAllMarkers”. DEGs between spatial regions were identified using “FindMarkers”, employing the Wilcoxon rank‐sum test with Bonferroni correction (|Log2(fold change)| > 0.25, adjusted p‐value < 0.05).

For scRNA‐seq analysis, normalization was performed using the “NormalizeData” function, while the remaining analytical steps were similar to those applied to spRNA‐seq.

### Pathological Region Annotation and Niche Identification

For pathological annotation, spRNAseq analysis was first performed independently for each sample. Based on unsupervised clustering results and the spatial morphology of tissue sections, four major pathological regions, including ATC, HGFCTC, DTC, and stroma, were collaboratively annotated by two board‐certified pathologists. The boundaries of each region were further refined to ensure consistency with histological diagnosis.

For niche annotation, after batch effect correction using the Harmony algorithm, a total of 16 spatial niches were identified across all samples. Each niche was annotated based on two criteria: (a) its spatial distribution relative to the pathologist‐defined regions and (b) the expression patterns of marker genes that distinguish between niches within the same pathological region. The detailed workflow is illustrated in Figure S5A (Supporting Information).

### Gene Ontology (GO) and Gene Set Variation Analysis (GSVA) Analysis

GO analysis was conducted using the Metascape online platform (www.metascape.org). GO term p‐values were computed based on the cumulative hypergeometric distribution within Metascape. For each cell type, only 5 to 10 disease‐associated GO pathways were selected for visualization. GSVA, an unsupervised gene set enrichment approach designed to evaluate pathway activity changes at the individual sample level, was performed using the GSVA package (v1.48.3) in R. The results of the pathway analysis were visualized using the pheatmap (v1.0.12) and ggplot2 (v3.2.1) packages.

### Pseudotime Trajectory Analysis

To infer dedifferentiation trajectories, both Monocle3 (v1.3.1) and Slingshot (v2.6.0) were employed for cross‐validation and complementary insights. For Monocle3, metadata and the top 2000 highly variable genes were imported from the integrated Seurat object. Niches were re‐clustered, and trajectories were constructed using the learn_graph function. Genes with q‐value < 0.01 and Moran's I > 0.2 (identified via graph_test) were used to order cells in pseudotime. Genes dynamically expressed along pseudotime were also identified using graph_test. For Slingshot, UMAP embeddings and cluster labels of niches were extracted from the Seurat object. Lineage reconstruction and pseudotime estimation were performed using principal curves, allowing identification of branching trajectories and key bifurcation points.

### Cell2location

Cell2location was used to integrate scRNA‐seq and spRNA‐seq to infer the spatial distribution of cell populations in thyroid carcinoma tissues. The analysis followed the standard pipeline proposed by its developers.^[^ ^58^ ^]^ High‐quality scRNA‐seq data from ATC and DTC were used as a reference to ensure comprehensive representation of cellular subpopulations. Cell2location mapped single‐cell‐defined cell types onto spatial transcriptomic spots, leveraging scRNA‐seq priors to estimate posterior distributions while correcting for technical and biological variability. Bayesian inference ensured accurate spatial mapping of cell types.

### Laser Capture Microdissection and Whole‐Exome Sequencing (WES)

Laser capture microdissection was performed on ATC mixed with DTC specimens, with tissue dissection conducted by experienced pathologists to isolate thyroid carcinoma regions with different degrees of differentiation. Genomic DNA was extracted from thyroid carcinoma tissue and quantified using a Qubit fluorometer. DNA integrity and fragment size were assessed with an Agilent 2100 Bioanalyzer. Libraries were prepared using the Agilent SureSelect XT Human All Exon V7 kit, following enzymatic fragmentation, end repair, A‐tailing, and adapter ligation. Target exonic regions were captured with biotin‐labeled probes and enriched using streptavidin magnetic beads.

Final libraries were amplified, purified, and sequenced on the Illumina NovaSeq 6000 platform (150 bp paired‐end). Quality control was performed with FastQC, and low‐quality reads were removed using Trimmomatic. Reads were aligned to the human genome (GRCh38) with BWA‐MEM, and duplicate reads were marked with Picard Tools. Variants were called using GATK HaplotypeCaller and annotated with ANNOVAR, filtering high‐confidence variants based on sequencing depth, allele frequency, and functional impact to identify potential driver mutations in thyroid carcinoma.

### Cell Culture

Tumor cells were cultured in a sterile environment using DMEM or RPMI‐1640 supplemented with 10% FBS and 1% penicillin/streptomycin. Cells were maintained at 37 °C in a 5% CO_2_ incubator. The cell lines used in this study included human DTC cell lines (TPC‐1, B‐CPAP, K1, KTC‐1) and human ATC cell lines (BHT101, ACT‐1, 8305C). The mouse ATC cell line Mouse thyroid‐BRAF/P53 Cre (BPC) was kindly provided by Professor Yulong Wang from Fudan University Shanghai Cancer Center.^[^ ^17^, ^59^ ^]^ All cell lines were authenticated by STR profiling and tested negative for mycoplasma contamination.

### Plasmid Construction and Lentivirus Packaging

Total RNA was extracted from 293T cells and reverse transcribed into cDNA. The target gene coding sequence was amplified using specific primers and inserted into the pLex‐MCS plasmid via BamH I/Nhe I digestion and ligation. Synthesized sgRNA fragments were phosphorylated with T4 PNK, annealed, and ligated into the BsmB I‐digested lentiCRISPR v2 plasmid.

The constructed lentiviral plasmid was co‐transfected into 293T cells with pMD2.G and PSPAX2 packaging plasmids. Supernatants were collected at 48 h post‐transfection. Cells underwent three freeze‐thaw cycles to ensure complete lysis, followed by centrifugation to remove debris. Viral particles were enriched via ultracentrifugation and either used immediately for infection or stored at −80 °C for future use.

### Establishment of Stable Cell Lines

The cell line was infected with the lentivirus in the presence of polybrene at a final concentration of 8 µg mL^−1^. After 12 h of infection, the medium was replaced with fresh culture medium. Cells were then cultured for an additional 72 h and subjected to selection with 1 µg mL^−1^ puromycin until no further cell death was observed. The establishment of stable cell lines was confirmed using WB and qPCR analyses.

### In Vitro Induction and Co‐Culture of M2 Macrophages

For human experiments, THP‐1 cells were first cultured to an appropriate density. Cells were then stimulated with phorbol 12‐myristate 13‐acetate (PMA, 100 nM) for 48 h to induce adherence and differentiation into M0 macrophages. After a 24‐h resting period, cells were further stimulated with interleukin‐4 (IL‐4, MCE, HY‐P70653, 20 ng ml^−1^) and interleukin‐13 (IL‐13, MCE, HY‐P72595, 20 ng ml^−1^) for an additional 48 h to promote polarization toward the M2 phenotype. For mouse experiments, RAW 264.7 cells, as M0‐type macrophages, were directly stimulated with IL‐4 and IL‐13 to induce polarization toward the M2 phenotype.

For co‐culture experiments, conditioned media (supernatants) from different treatment groups (e.g., PDCD4 overexpression versus vector control) were applied to the M0 macrophages to assess their ability to induce M2 polarization. The expression of M2 macrophage markers was subsequently evaluated by flow cytometry to determine the effect of the different supernatants on M2 phenotype induction (Figure S13A, Supporting Information).

### Mouse Subcutaneous Tumor Model

All animal experiments were conducted in accordance with the guidelines approved by the Animal Ethics Committee of SYSUCC (L025503202412015). This study used SPF‐grade 6–8‐week‐old female C57 mice, housed and handled in an SPF animal facility. For the subcutaneous tumor model, thyroid cancer cells were resuspended in PBS at a concentration of 1 × 10⁶ cells mL^−1^, and 100 µL of the suspension was injected subcutaneously. Tumor volume was measured with calipers every three days starting on day 7 post‐injection, using the formula V = 1/2 × length × width^2^. Once tumors reached 100 mm^3^, treatment was administered, and tumor growth was continuously monitored. The eIF4A inhibitor CR‐1‐31‐B (MCE, Cat#HY‐136453) was administered to mice at a dose of 2 mg kg^−1^ via intraperitoneal injection every other day in mouse drug administration experiments. The control group received the same proportion of DMSO and normal saline, administered via the same route, at the same frequency, and starting at the same time as the treatment group. After three weeks of treatment, mice were euthanized, and tumor samples were collected, photographed, and fixed in 4% paraformaldehyde for paraffin embedding and further analysis.

### Preparation of Single‐Cell Suspension

Tumor tissues were rinsed with HBSS (Gibco), minced, and digested at 37 °C for 30 min with 25 µg mL^−1^ Liberase TM (Roche) and 50 µg mL^−1^ DNase (Sigma) in RPMI (Gibco) under gentle agitation. Simultaneously, mechanical dissociation was performed using the Tumor Cell Isolation Kit (Miltenyi, Cat#130110187) following the manufacturer's instructions. The digested tissue was passed through a 70 µm filter to obtain a single‐cell suspension, and excess red blood cells were removed using RBC lysis buffer.

### Flow Cytometry (FCM)

For FCM staining of macrophages, co‐cultured THP‐1 cells or mouse tumor‐derived single‐cell suspensions were incubated at 37 °C and stimulated for 5 h with 5 ng mL^−1^ phorbol 12‐myristate 13‐acetate (PMA; Sigma‐Aldrich), 1 µg mL^−1^ brefeldin A (Sigma‐Aldrich), and 500 ng mL^−1^ ionomycin (Sigma–Aldrich). Following stimulation, cells were initially stained with live/dead viability dyes and surface antibodies, followed by permeabilization, fixation, and intracellular antibody staining. Stained cells were acquired using a flow cytometer and analyzed with FlowJo(version 10.0.7, BD Biosciences). For co‐cultured THP‐1 cells, cells were stained with the following antibodies: CD11b‐APC (BioLegend Cat#301310), CD206‐BV421 (BioLegend Cat#321126), and CD163‐FITC (BioLegend Cat#333618). For mouse tumor‐derived single‐cell suspensions, cells were stained using the following antibodies: CD11b‐FITC (BioLegend Cat#101206), CD80‐APC (BioLegend Cat#141708), F4/80‐PE (BioLegend Cat#123110), IL‐10‐BV421(BioLegend Cat#505022), CD163‐APC (BioLegend Cat#155306), and TNF‐BV650 (BD Biosciences Cat#563943).

### Protein Extraction and Western Blotting (WB)

Cells were lysed using RIPA buffer (Beyotime, P0013B) supplemented with a protease inhibitor cocktail Ι (Calbiochem, San Diego, CA). The protein extracts were resolved by SDS‐PAGE (10% or 12.5% polyacrylamide, EpiZyme, Shanghai) and subsequently transferred onto polyvinylidene difluoride membranes (Roche). After blocking with 5% skimmed milk in Tris‐Buffered Saline with Tween (TBST), the membranes were incubated overnight at 4 °C with primary antibodies. Following four washes with TBST (10 min each), membranes were exposed to secondary antibodies (Beyotime, Shanghai, China) for 1 h at room temperature. After three additional washes, protein bands were visualized using an enhanced chemiluminescence detection system (Tanon, Shanghai, China) and captured with the ChemiDoc Touch Imaging System (BIO‐RAD, Hercules, CA). The antibodies used for WB included: PDCD4 (Proteintech Cat#12587‐1‐AP), GAPDH (Proteintech Cat# 60004‐1‐Ig), eIF4A (Cell Signaling Technology Cat#2013), eIF4E (Cell Signaling Technology Cat#2067), eIF4G (Cell Signaling Technology Cat#2469).

### Multiplex Immunofluorescence (MIF) and Immunohistochemistry (IHC)

To evaluate the expression of specific proteins, MIF or IHC analysis was performed on FFPE tumor specimens. The slides were first deparaffinized using an eco‐friendly clearing agent, followed by sequential rehydration with graded ethanol. Endogenous peroxidase activity was blocked using 10% hydrogen peroxide, and antigen retrieval was carried out via microwave treatment.

For IHC, the slides were incubated with the primary antibody at 4 °C for 16 h. After washing, an enzyme‐conjugated goat anti‐mouse/rabbit IgG polymer was applied and incubated for 1 h, followed by DAB staining and hematoxylin counterstaining. The evaluation of IHC was performed by experienced pathologists. The antibodies used for IHC included: PDCD4 (Proteintech Cat#12587‐1‐AP), CD163 (OriGene Cat#TA506386), CD20 (Cell Signaling Technology Cat# 48750), CD4 (Proteintech Cat#67786‐1‐Ig), and CD8 (ZSGB‐Bio, Cat#ZA‐0508).

For MIF, staining was conducted according to the manufacturer's instructions for the MIF kit (Panovue, Cat#10002100100). Briefly, after antigen retrieval, slides were incubated with the primary antibody at room temperature for 1 h, followed by the addition of an HRP‐conjugated secondary antibody solution. Fluorescent staining was achieved using a signal amplification fluid diluted with a fluorescent dye. For each antibody, a new round of antigen retrieval, primary antibody incubation, secondary antibody staining, and fluorescent dye application was performed. Nuclei were counterstained with DAPI, and slides were mounted using an antifade mounting medium. The stained slides were scanned, and quantitative analysis was performed using HALO software (Indicalab, New Mexico, USA). The antibodies used for MIF included: TYMP (Proteintech Cat#12383‐1‐AP), CD163 (OriGene Cat#TA506386).

### RNA Extraction and Quantitative Polymerase Chain Reaction (qPCR)

Cells or tissue samples were lysed in an appropriate volume of TRIzol reagent (Invitrogen, Cat#15596026) and homogenized thoroughly. After incubation at room temperature for 5 min to ensure complete cell lysis and RNA release, chloroform was added and vigorously shaken. Following phase separation by centrifugation, the aqueous phase was collected, and an equal volume of isopropanol was added to precipitate RNA. After a 10‐min incubation at room temperature, samples were centrifuged again, the supernatant discarded, and the RNA pellet washed with 75% ethanol. The purified RNA was dissolved in DEPC‐treated water, and RNA concentration and purity were assessed using a NanoDrop spectrophotometer. Throughout the procedure, RNase contamination was avoided, and all steps were performed under low‐temperature conditions to preserve RNA integrity. QPCR was conducted using the ABI Prism 7500 Fast Sequence Detection System (Applied Biosystems) with ChamQ SYBR qPCR Master Mix (Q311‐02, Vazyme). The sequences of gene‐specific primers are provided in Table S7 (Supporting Information).

### Protein Mass Spectrometry Analysis

Proteins were extracted from cells or supernatant samples, and concentrations were determined using the BCA assay. Protein digestion was carried out using the filter‐aided sample preparation (FASP) method. Briefly, proteins were reduced with 5 mM dithiothreitol (DTT) at 56 °C and alkylated with 15 mM iodoacetamide (IAA) in the dark at room temperature. Samples were sequentially washed with urea and HEPES buffers, followed by digestion with trypsin at enzyme‐to‐protein ratios of 1:50 (overnight) and 1:100 (4 h) at 37 °C. Peptides were desalted using Strata‐X columns, and concentrations were measured using the Pierce Quantitative Peptide Assay Kit (Thermo Scientific).

Peptides were separated on a Vanquish Neo nanoflow LC system (Thermo Scientific) and analyzed using an Astral high‐resolution mass spectrometer in data‐independent acquisition (DIA) mode. MS1 spectra were acquired over an m/z range of 380–980 with a resolution of 240000. DIA acquisition was performed using 299 windows with a 2 m/z isolation width and a normalized collision energy of 25 eV, optimized for high‐throughput quantification. Raw DIA data were processed using DIA‐NN software, and protein identifications were filtered using a false discovery rate (FDR) of <1%.

### Polysome Profiling Analysis

Cycloheximide was added to the culture medium at a final concentration of 100 µg mL^−1^, and cells were incubated at 37 °C for 10 min to inhibit translation. Cells were then placed on ice and washed twice with ice‐cold PBS before being lysed in lysis buffer. The lysates were gently sheared and centrifuged at 12000 × g for 10 min at 4 °C. The supernatant was collected for further analysis or stored at −80 °C. Linear sucrose gradients (10–50%) were prepared using a gradient mixer and pre‐chilled at 4 °C for 45 min before sample loading. Ultracentrifugation was performed using an SW‐41 rotor at 36000 rpm for 2 h at 4 °C. Gradients were then fractionated using a gradient fractionator, and RNA‐containing fractions were collected for downstream analyses.

### Statistics

All experiments were conducted at least three times. Data analysis and visualization were performed using GraphPad Prism software (version 8.0.2, GraphPad, Inc., La Jolla, CA, USA) and R software (version 4.3.2). Results are expressed as mean ± standard error of the mean (SEM). Statistical comparisons were performed using one‐way ANOVA, and paired or unpaired t‐tests. Prognostic analysis was conducted using the log‐rank test. A p‐value of less than 0.05 was considered statistically significant. ns denotes no significance; ^*^ *p* < 0.05; ^**^ *p* < 0.01; ^***^ *p* < 0.001; ^****^ *p* < 0.0001.

## Conflict of Interest

The authors declare no conflict of interest.

## Supporting information



Supporting Information

Supporting Information

## Data Availability

The human spatial RNA sequencing data are available in the Genome Sequence Archive (GSA) at the National Genomics Data Center, Beijing Institute of Genomics, Chinese Academy of Sciences (https://ngdc.cncb.ac.cn/gsa‐ human/, HRA010818). The raw experimental data generated in this study have been deposited in the National Scientific Data Sharing Platform (https://www.researchdata. org.cn/, RDDB2025362384). All scripts used for data processing and statistical analysis are available in our GitHub repository (https://github.com/Nick‐ningkang/ spRNAseq‐analysis‐of‐ATC‐coexisting‐with‐DTC). The single‐cell RNA sequencing data were obtained from publicly available datasets, including GSE148673, GSE184362, GSE193581, and GSE210347. Bulk transcriptomic cohorts for thyroid cancer were obtained from GSE33630 and The Cancer Genome Atlas (TCGA). Other data that support the findings of this study are available from the corresponding author upon reasonable request.
