# Supplementary material for: Spatial Transcriptomics Reveals Transcriptomic and Immune Microenvironment Reprogramming during Thyroid Carcinoma Dedifferentiation
Source: Adv Sci (Weinh). 2025 Sep 4;12(44):e06925. doi: 10.1002/advs.202506925 (PMC12667454; doi:10.1002/advs.202506925)
Supplement: Supplementary file 1 — Supporting Information [file ADVS-12-e06925-s002.pdf]

Figure S1

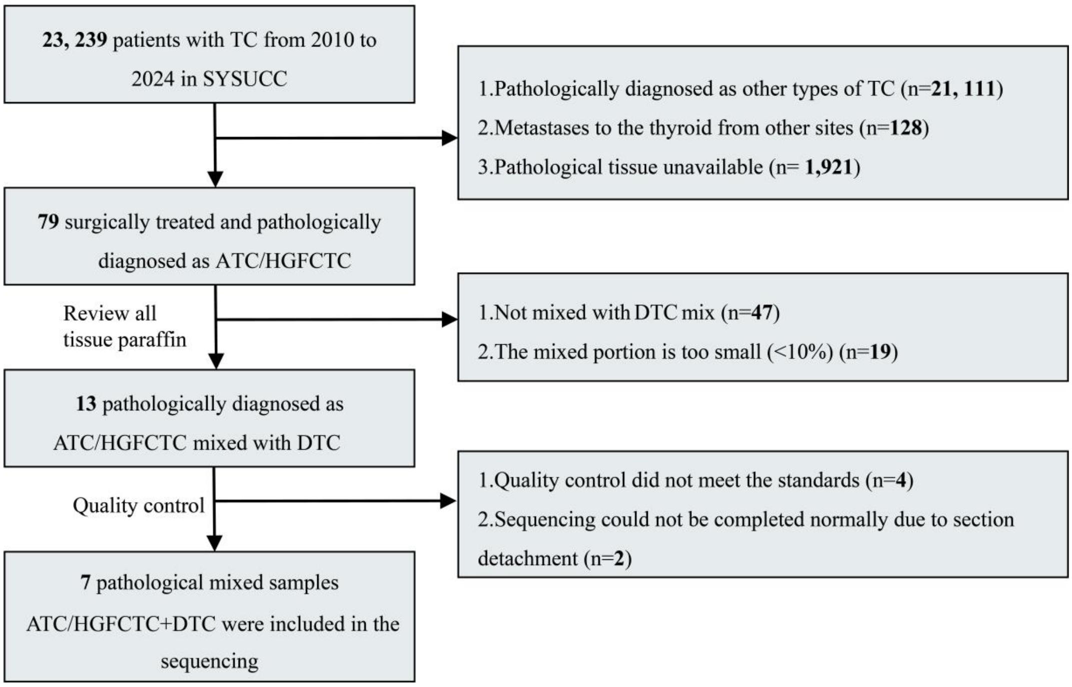

Figure S1. Flow chart of sample selection.

TC: Thyroid Cancer; **SYSUCC**: Sun Yat-sen University Cancer Center; **ATC**: Anaplastic Thyroid Cancer; **HGFCTC**: High-Grade Follicular Cell-derived Thyroid Carcinoma. **DTC**: Differentiated thyroid carcinoma.

Figure S2

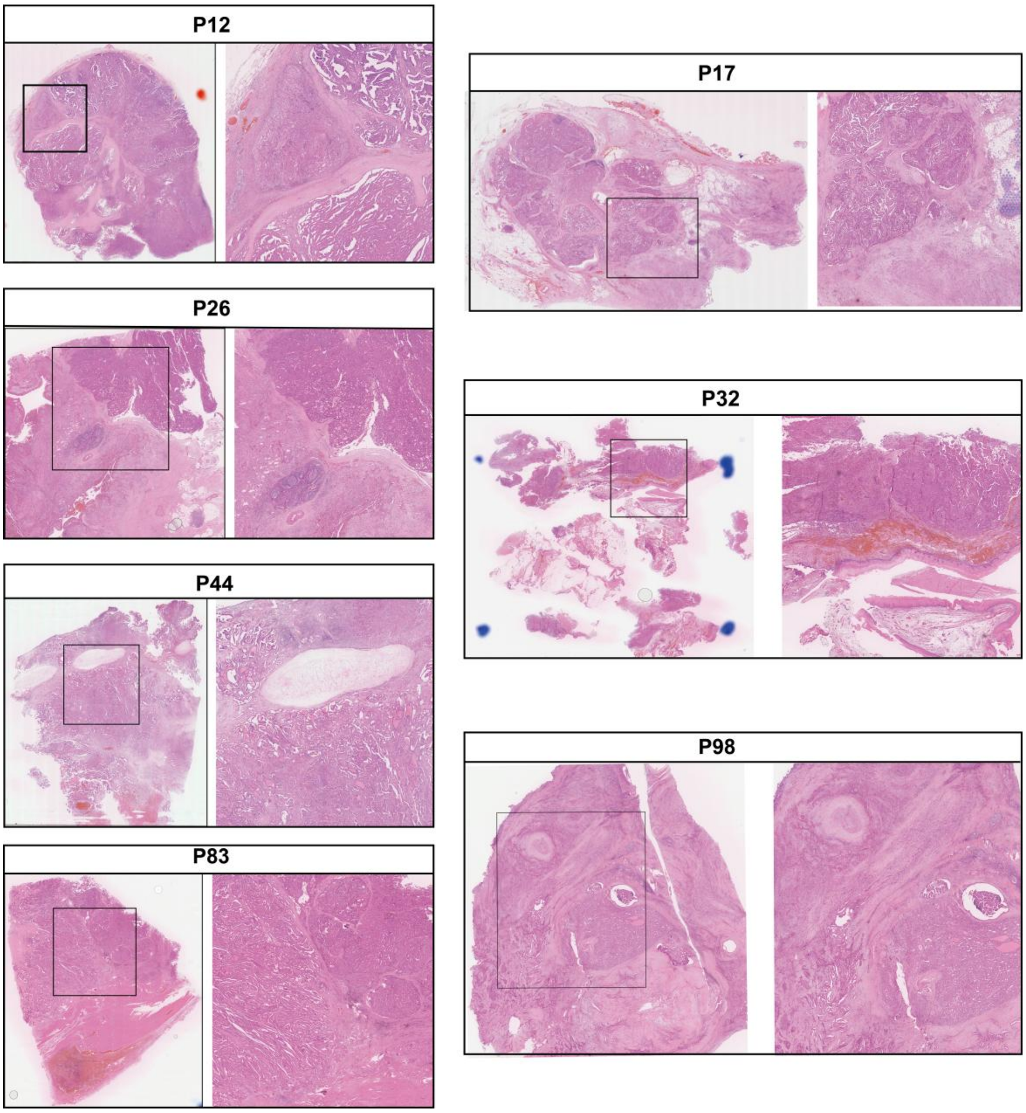

Figure S2. Diagram of spRNA-seq Regions on Pathological Slides of 7 ATC/PDTC-DTC coexisting samples.

ATC: Anaplastic thyroid cancer; **DTC**: Differentiated Thyroid Cancer; **PDTC**: Poorly Differentiated Thyroid Carcinoma; **spRNA-seq**: Spatial RNA Sequencing.

Figure S3

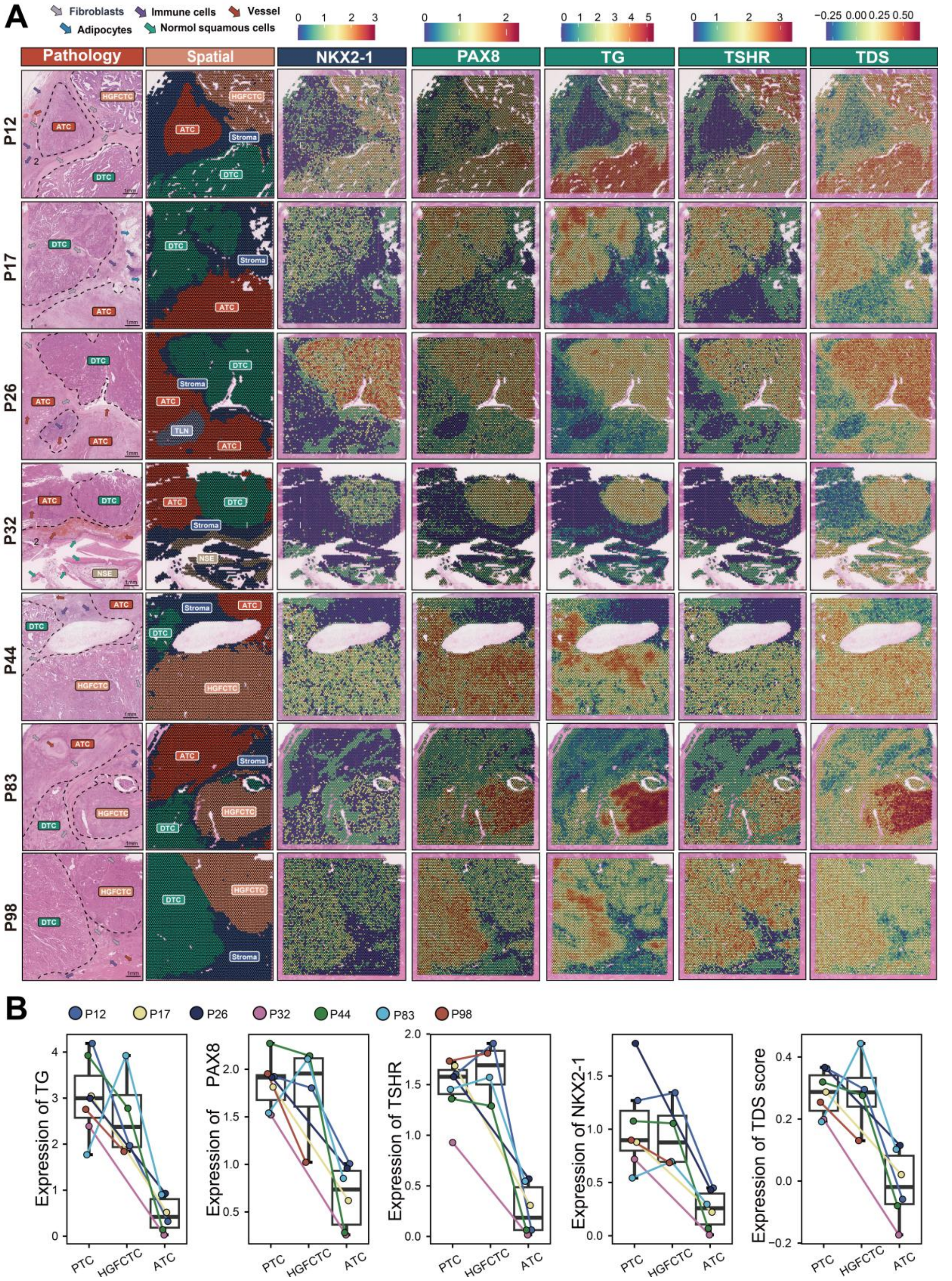

Figure S3. Analysis of TDS Scores and Differentiation-Related Genes in spRNAseq.

A. Visualization of TDS scores and differentiation-related genes in spRNAseq.

B. Boxplot showing expression levels of TDS scores and differentiation-related genes in different pathological regions.

ATC: Anaplastic thyroid cancer; **DTC**: Differentiated Thyroid Cancer;**HGFCTC**: High-Grade Follicular Cell-derived Thyroid Carcinoma; **TDS**: Thyroid Differentiation Score; **spRNAseq**: Spatial RNA Sequencing;

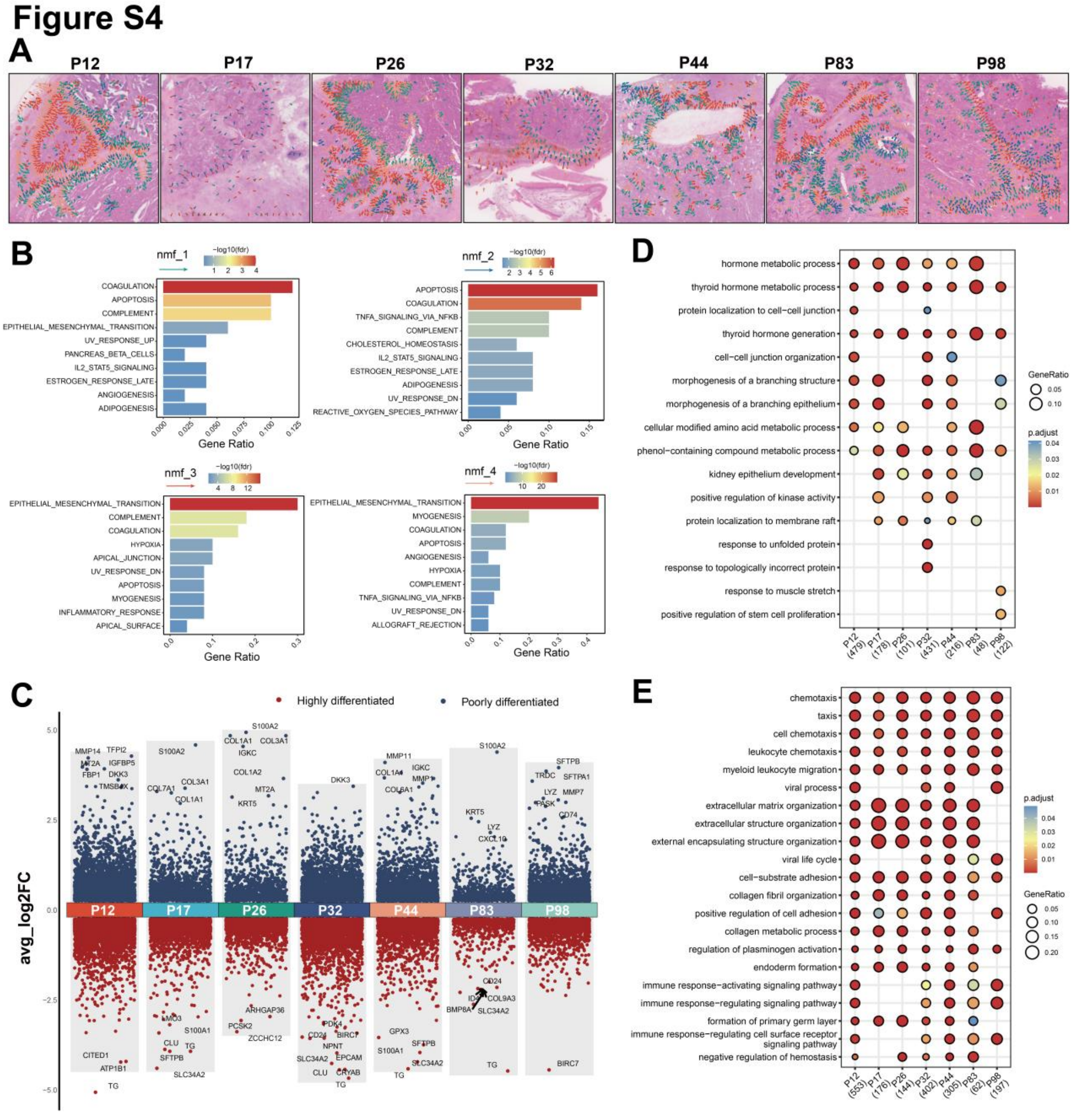

Figure S4. DEGs and Pathway Analysis of Single Sample from spRNAseq.

A. STG analysis reveals location-specific gene gradients.

B. Functional enrichment of top genes identified in STG analysis.

C. DEGs analysis of thyroid cancer with different differentiation grades in single samples.

D-E. GO enrichment analysis of downregulated (D) and upregulated (E) DEGs pathways.

DEGs: Differentially Expressed Genes; **spRNA-seq**: Spatial RNA sequencing; **STG**: Spatial Gene Gradient; **GO**: Gene Ontology.

Figure S5

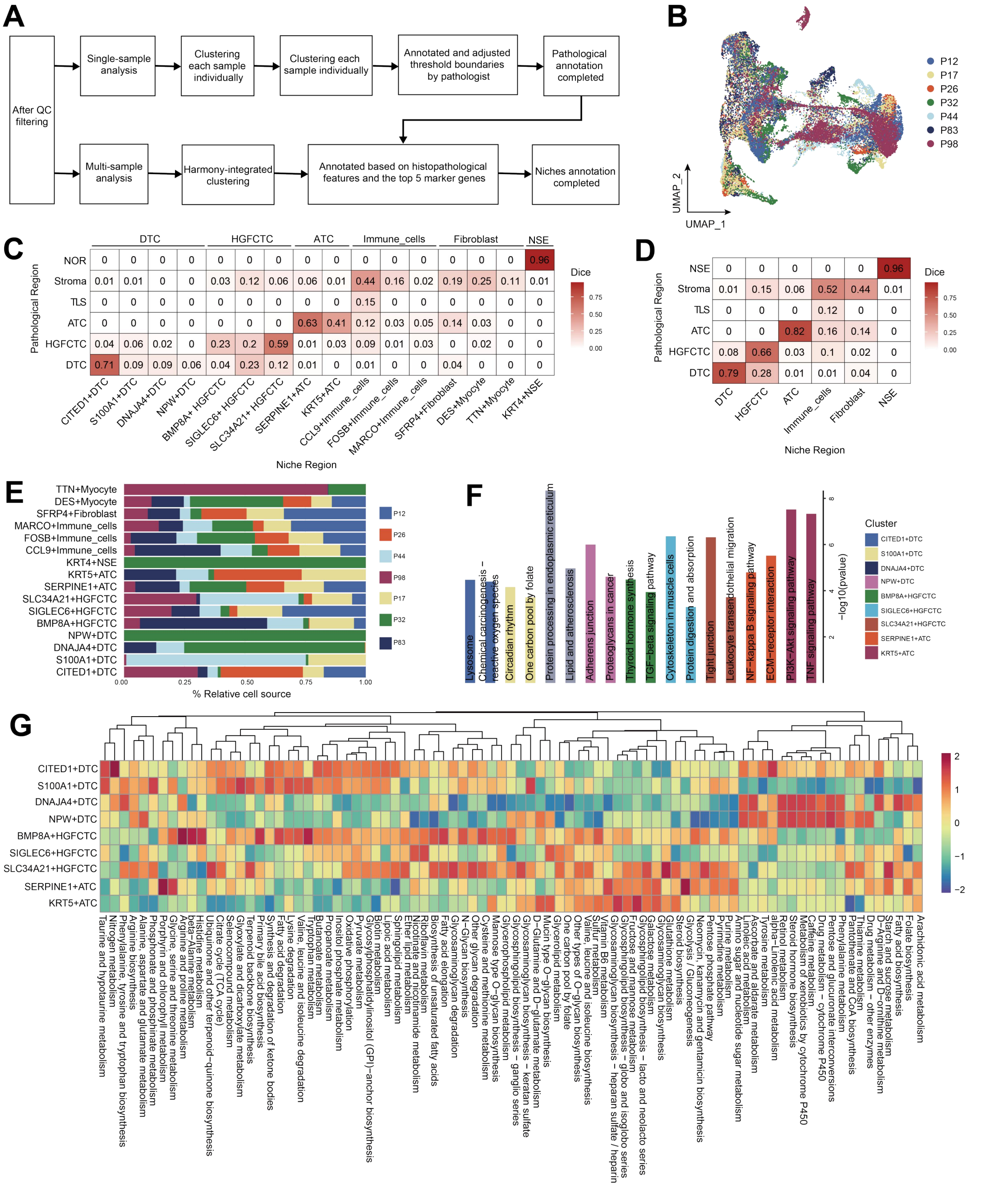

Figure S5. Multi-sample integration reveals tumor subtype composition and pathway characteristics.

A.Flowchart illustrating the annotation strategy of spatial niches in the multi-sample analysis.

B.UMAP plot showing spot distribution across individual samples.

C-D.Heatmaps of Dice coefficients comparing region annotations between single-sample pathology and multi-sample niche definitions.

E. Stacked bar plot illustrating the proportion of each cluster within different samples.

F. GO enrichment analysis of each tumor niche.

G. Metabolic pathway analysis of each tumor niche.

ATC: Anaplastic thyroid cancer; **DTC**: Differentiated Thyroid Cancer; **HGFCTC**: High-Grade Follicular Cell-derived Thyroid Carcinoma; **UMAP**: Uniform Manifold Approximation and Projection; **GO**: Gene Ontology; **GSVA**: Gene Set Variation Analysis

Figure S6

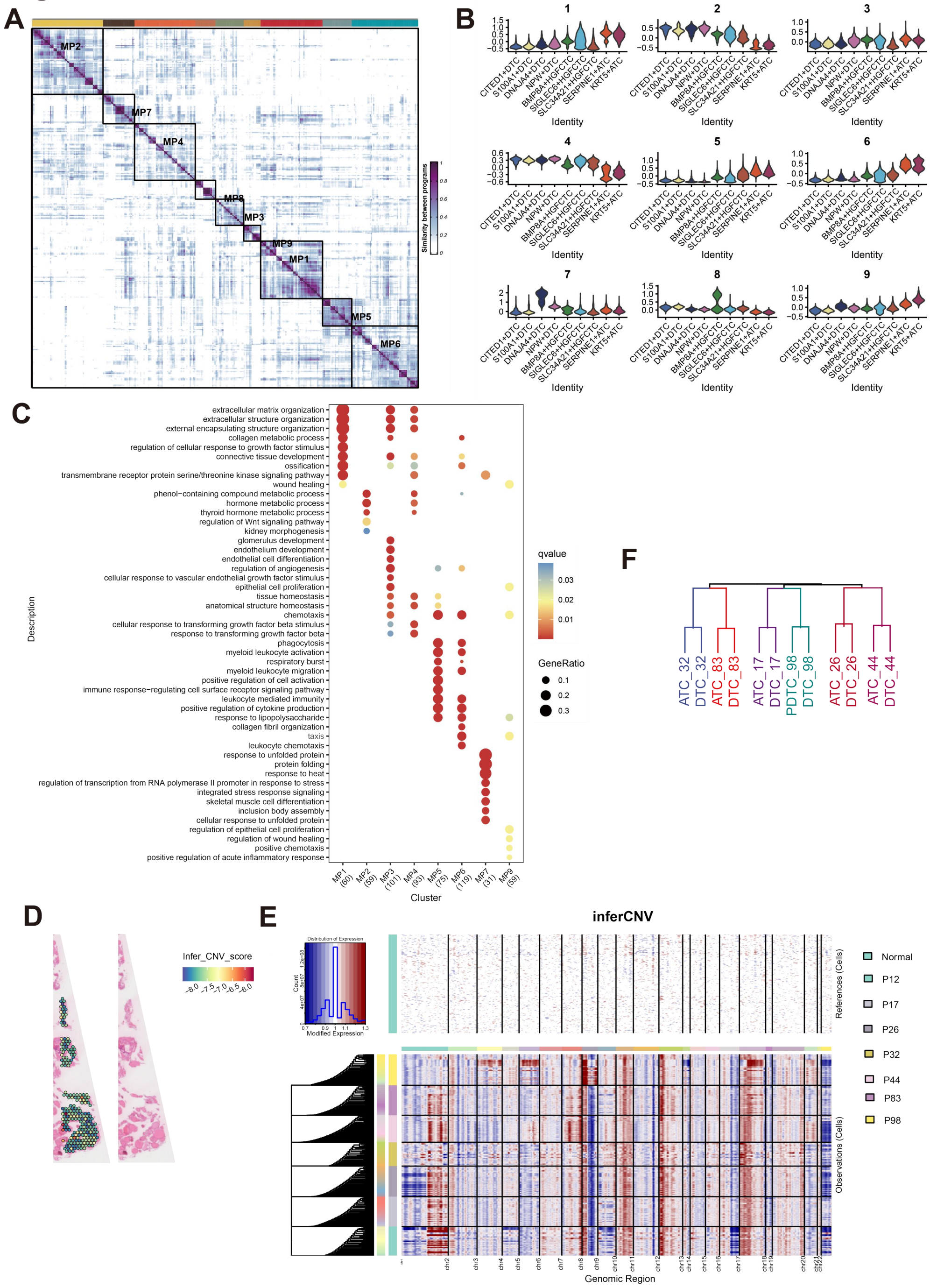

Figure S6. Tumor heterogeneity analysis based on spRNAseq and WES data.

A. NMF clustering identifies distinct transcriptional subtypes.

B. Proportional distribution of NMF subtypes across transcriptomic clusters.

C. Pathway enrichment analysis of NMF signature genes.

D. Normal thyroid epithelial cells used as a reference in inferCNV analysis.

E. inferCNV heatmap showing large-scale copy number variations across tumor regions.

F. Cluster analysis among WES profiles.

ATC: Anaplastic Thyroid Cancer; **DTC**: Differentiated Thyroid Cancer; **HGFCTC**: High-Grade Follicular Cell-derived Thyroid Carcinoma; **spRNA-seq**: Spatial RNA Sequencing; **WES**: Whole-exome Sequencing; **NMF**: Non-negative Matrix Factorization; **inferCNV**: Inference of Copy Number Variations.

Figure S7

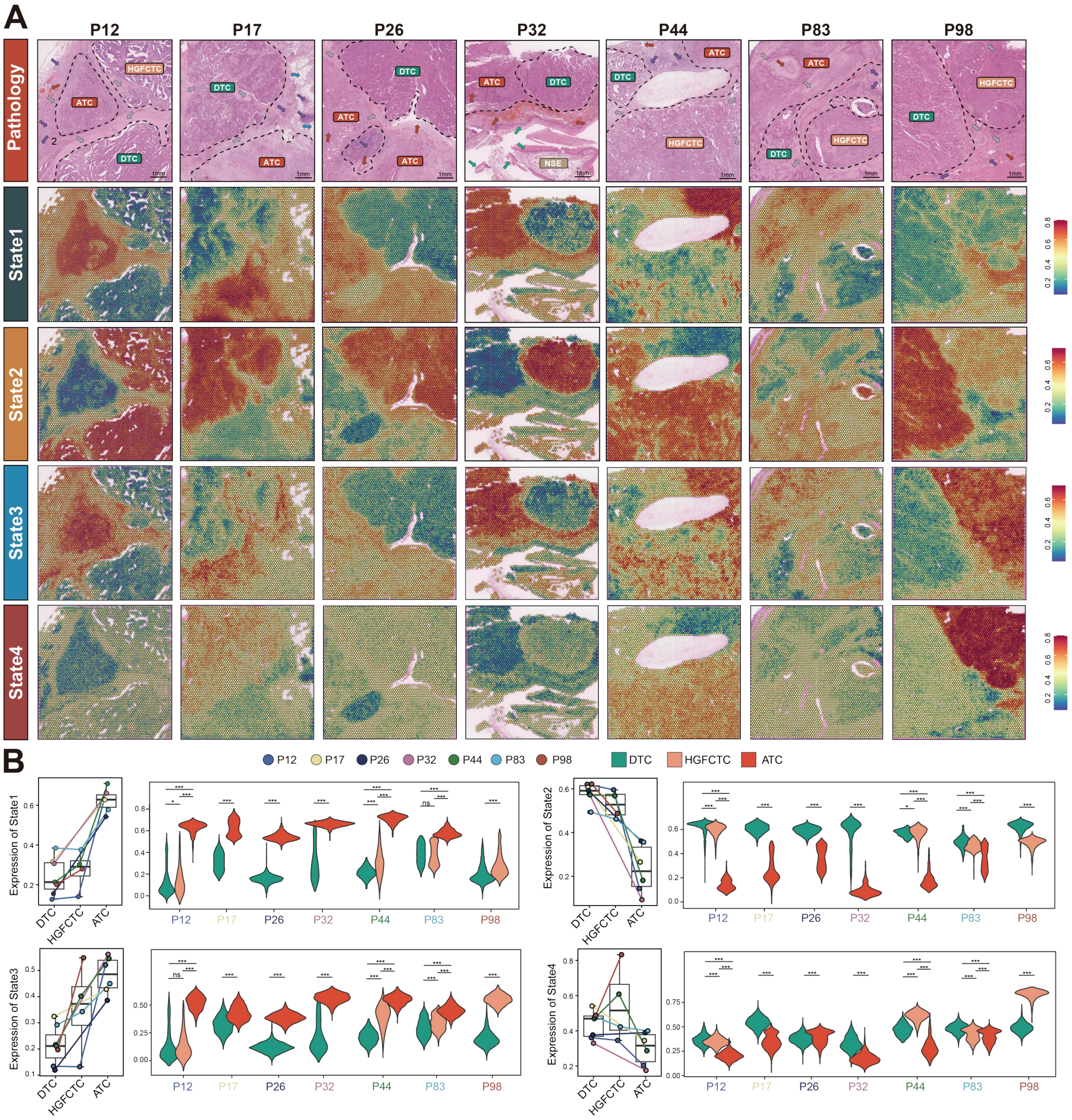

Figure S7. Distribution of Different State Scores in the spRNA-seq Landscape.

A. Visualization of various state scores in spRNA-seq data.

B. Boxplots (mean expression) and violin plots (single-sample) illustrating the distribution of state scores across different pathological regions.

spRNA-seq: Spatial RNA Sequencing; **ATC**: Anaplastic Thyroid Cancer; **HGFCTC**: High-Grade Follicular Cell-derived Thyroid Carcinoma. **DTC**: Differentiated thyroid carcinoma.

Figure S8

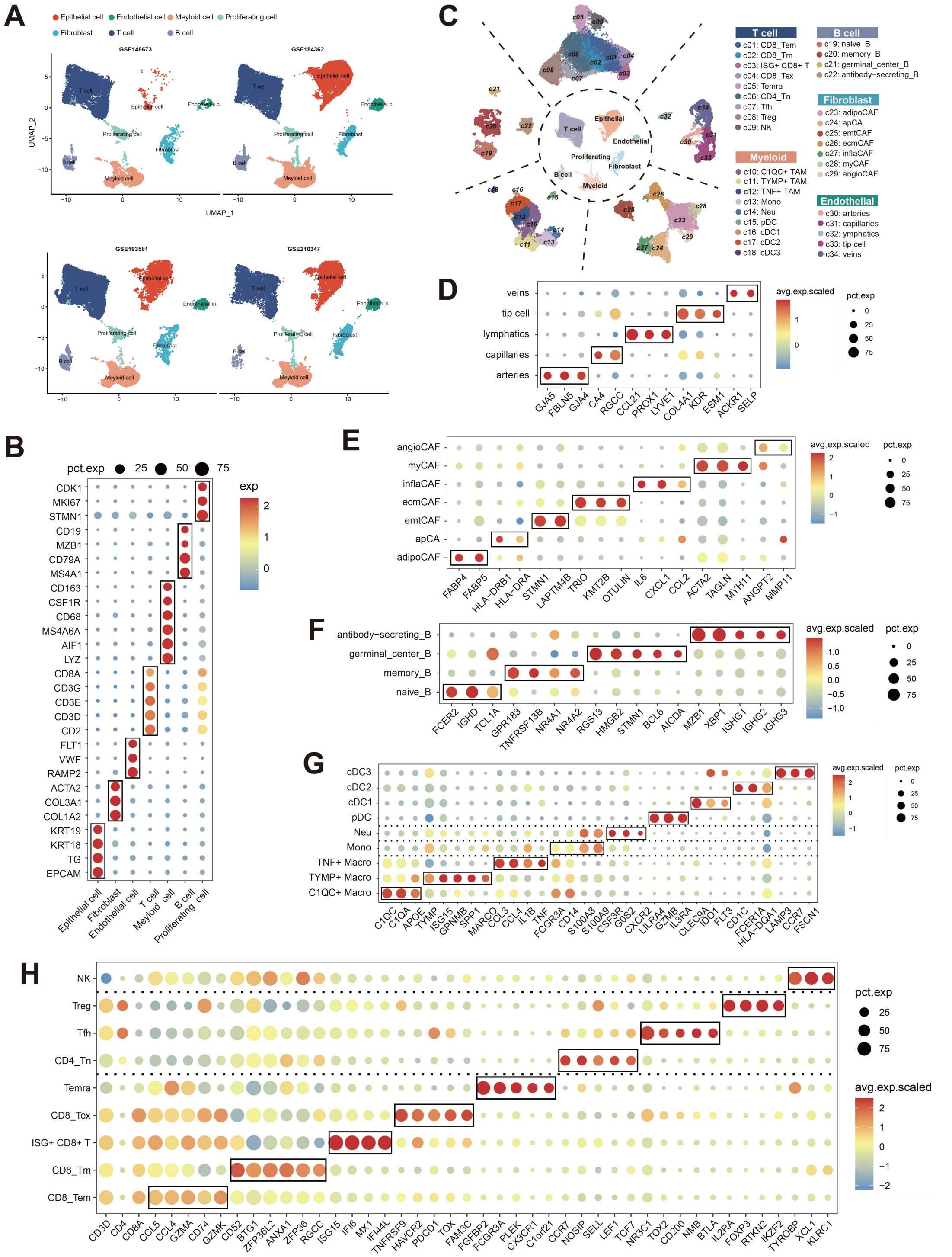

Figure S8. Annotation of cell subpopulations based on public scRNA-seq datasets

A. UMAP plots showing major cell clusters from different scRNA-seq datasets.

B. Marker gene expression profiles used to define major cell clusters.

C. Refined annotation of minor cell clusters in scRNA-seq.

D–H. Marker gene expression profiles used to define each minor cell clusters including endothelial cell (D), fibroblast (E), B cell (F), Myeloid cell (G), T cell (H).

scRNA-seq: Single-cell RNA sequencing; **UMAP**: Uniform manifold approximation and projection

Figure S9

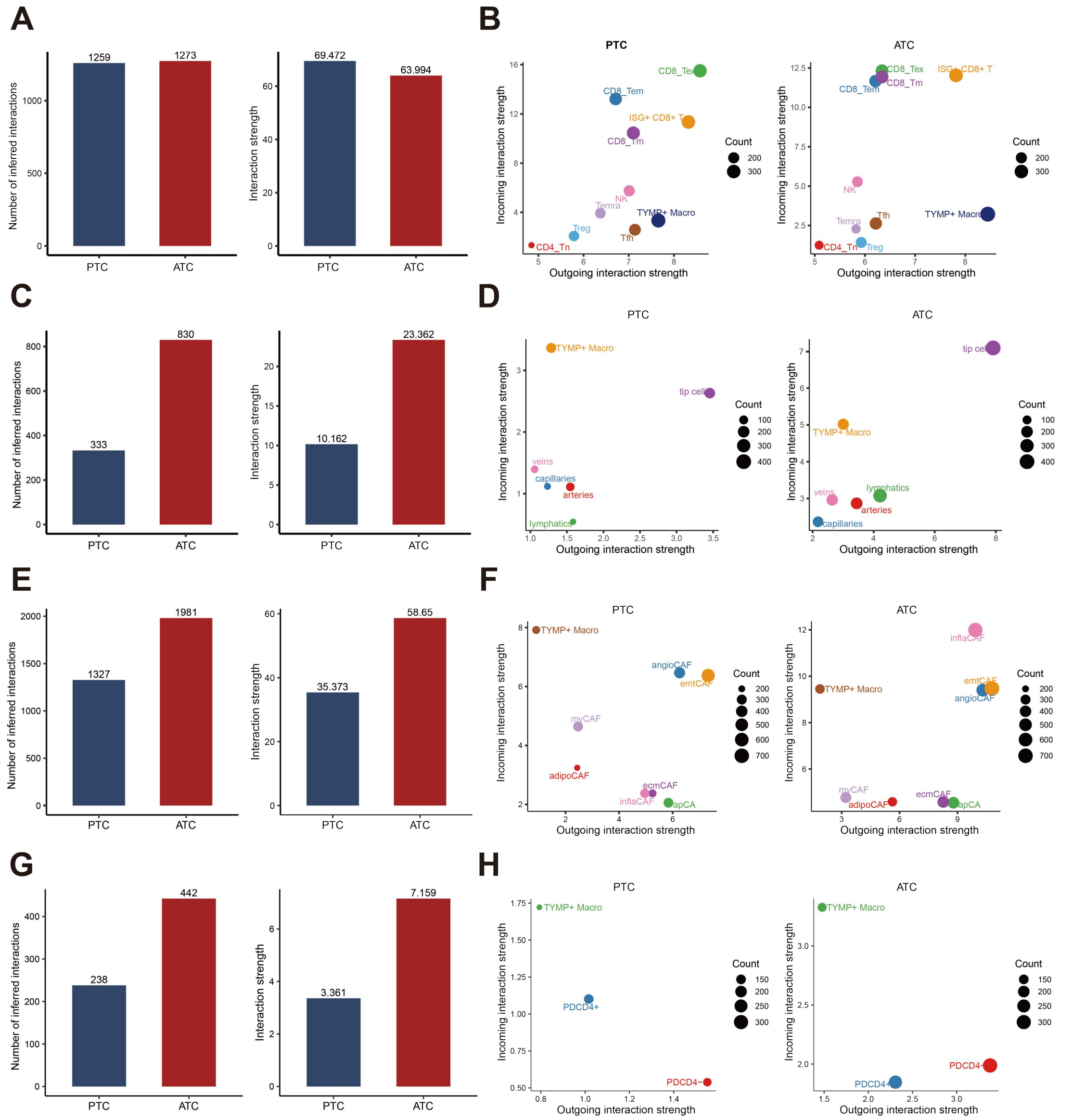

Figure S9. Analysis of cell–cell communication strength based on scRNAseq.

(A–B) Interaction strength between TYMP⁺ TAMs and CD8⁺ T cell subsets in PTC and ATC.

(C–D) Interaction strength between TYMP⁺ TAMs and endothelial cell subsets in PTC and ATC.

(E–F) Interaction strength between TYMP⁺ TAMs and fibroblast subsets in PTC and ATC.

(G–H) Interaction strength between TYMP⁺ TAMs and PDCD4⁺/⁻ tumor epithelial cells in PTC and ATC.

scRNA-seq: Single-cell RNA sequencing; **TYMP**: Thymidine phosphorylase;

TAMs: Tumor-associated macrophages; **PTC**: Papillary thyroid carcinoma;

ATC: anaplastic thyroid carcinoma; **PDCD4**: Programmed cell death protein 4.

Figure S10

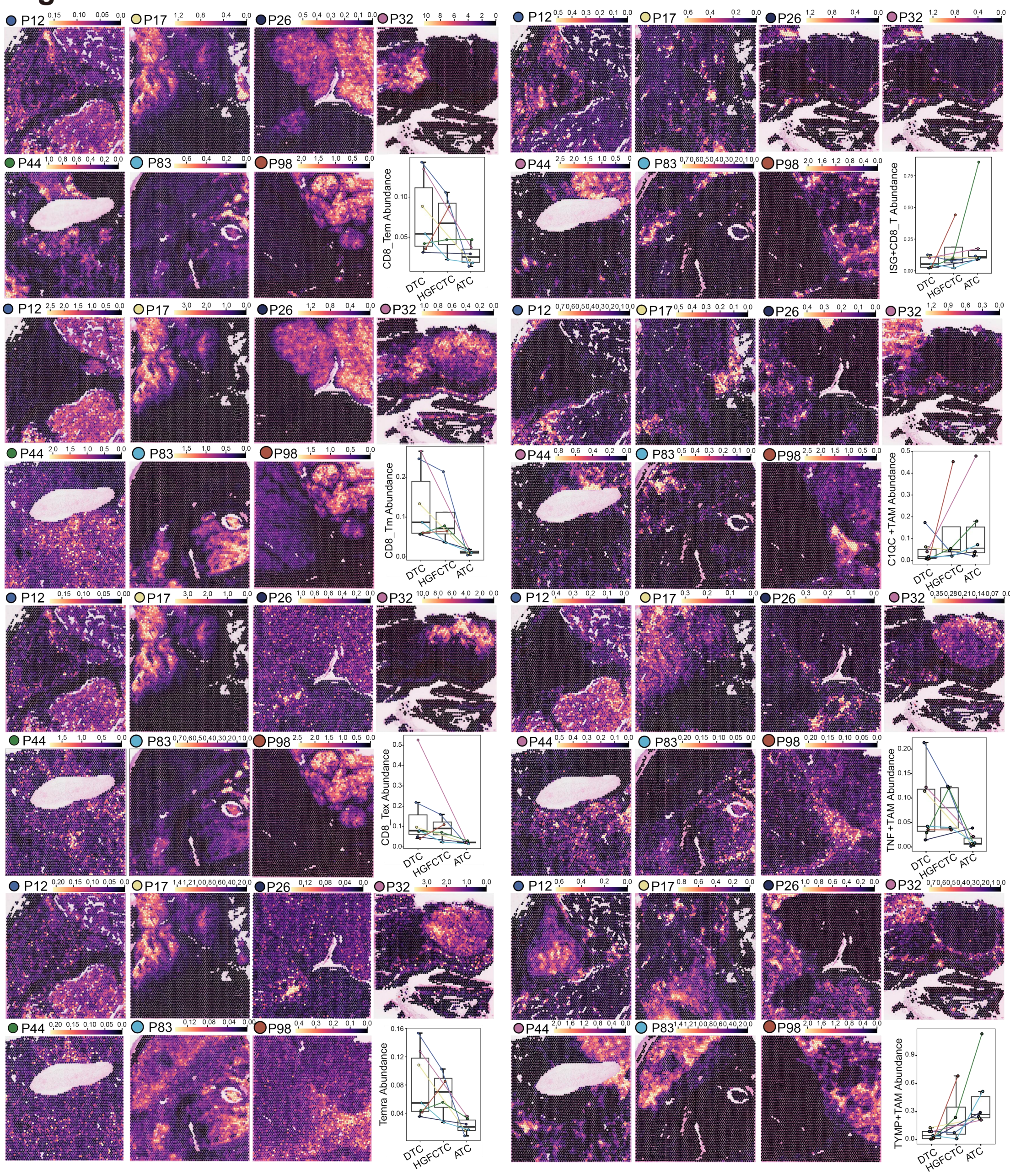

Figure S10. Spatial distribution of CD8⁺ T cell and myeloid subpopulations in the immune microenvironment of thyroid cancer revealed by single-cell analysis.

ATC: Anaplastic thyroid cancer; **DTC**: Differentiated Thyroid Cancer; **HGFCTC**: High-Grade Follicular Cell-derived Thyroid Carcinoma.

Figure S11

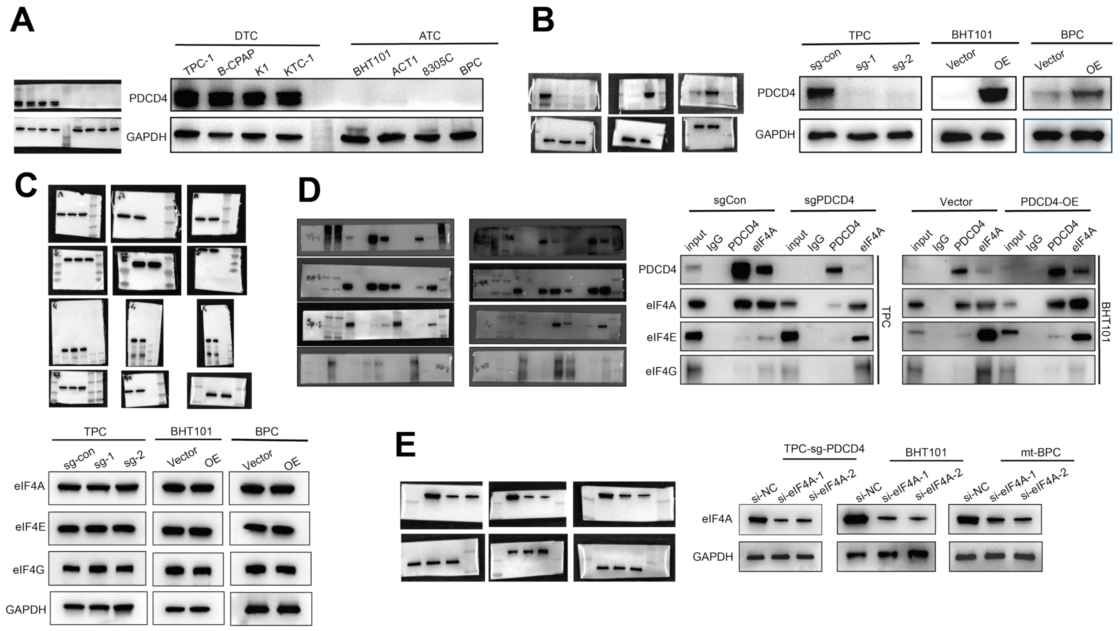

Figure S11. Original, unprocessed WB images

A. WB analysis of PDCD4 expression in thyroid cancer cell lines representing different histological subtypes.

B. WB validation of PDCD4 knockout in the DTC cell line (TPC-1) and PDCD4 overexpression in ATC cell lines (BHT101 and BPC).

C. WB analysis of eIF4A, eIF4E, and eIF4G expression levels in different groups with PDCD4 knockout or overexpression.

D. Co-IP analysis showing the changes in the eIF4F complex (eIF4A-eIF4E-eIF4G) upon PDCD4 knockout or overexpression in BHT101 cells.

E. WB analysis confirmed the knockdown efficiency of eIF4A using siRNA.

ATC: Anaplastic thyroid cancer; **DTC**: Differentiated Thyroid Cancer; **Co-IP**: Co-immunoprecipitation; **WB**: Western Blot;

Figure S12

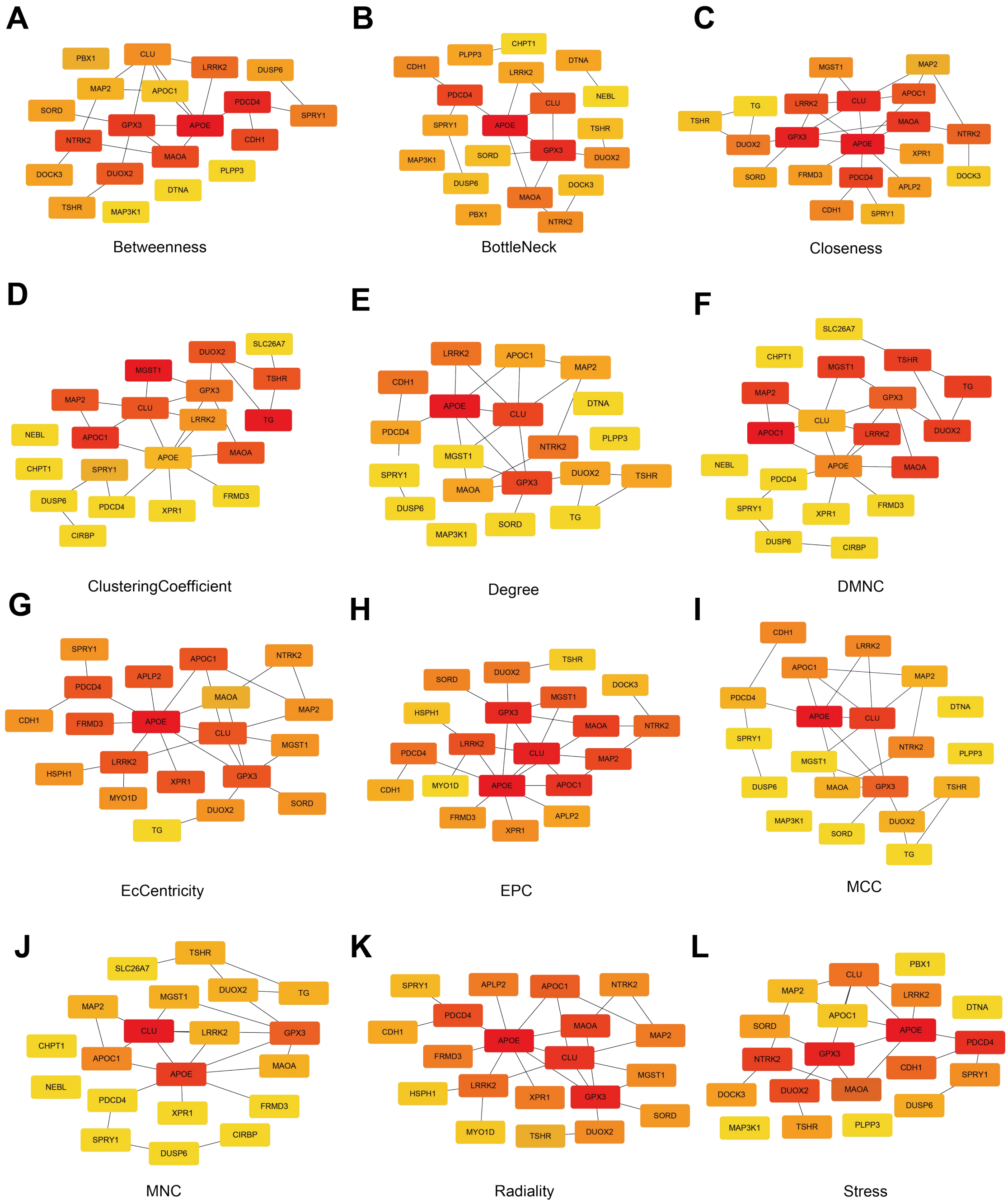

Figure S12. Identification of hub genes using different algorithms in Cytoscape. DMNC: Density of Maximum Neighborhood Component; EPC: Edge Percolated Component; MCC: Maximal Clique Centrality; MNC: Maximum Neighborhood Component.

Figure S13

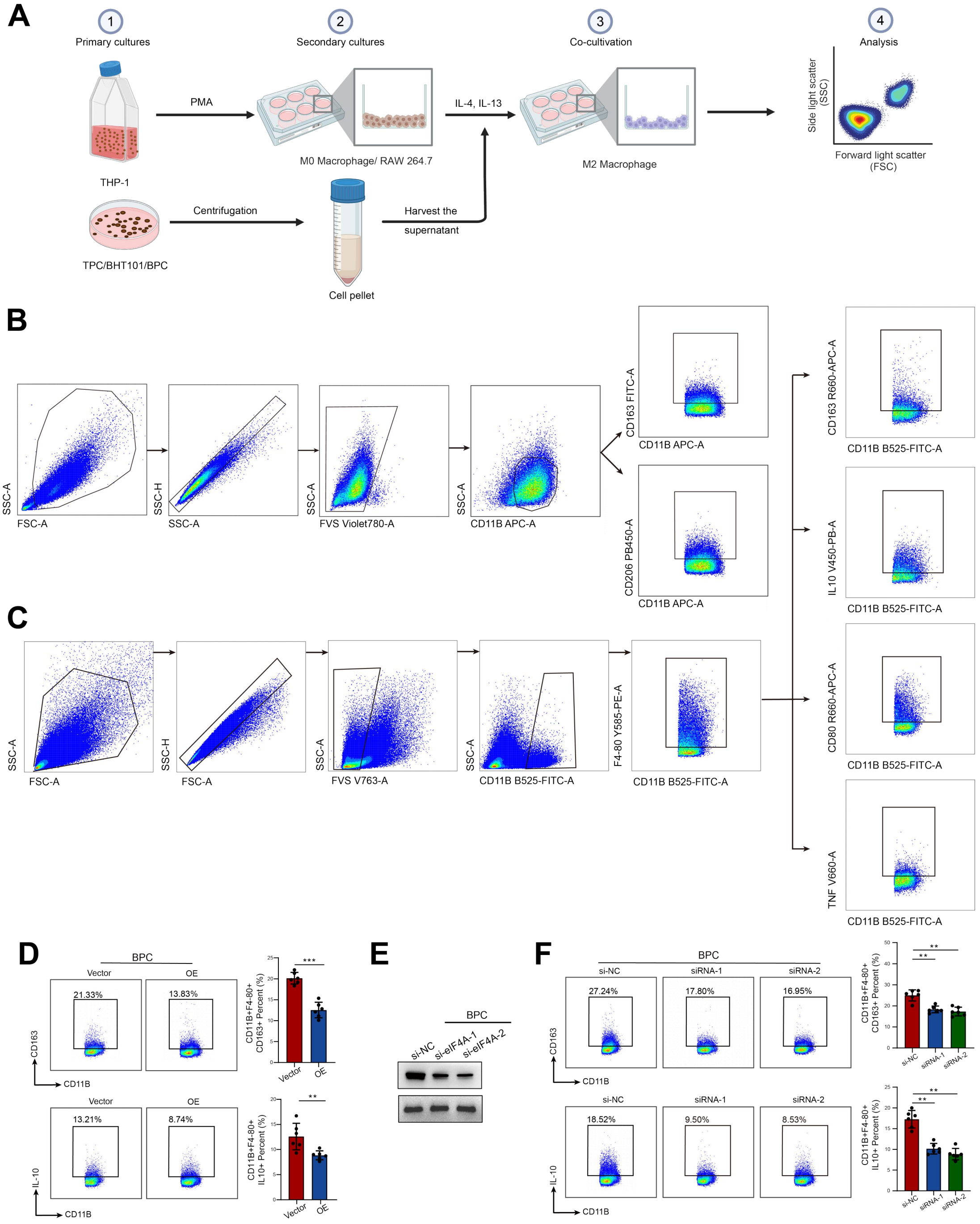

Figure S13.FCM gating strategies used in this study.

A.Flowchart of the THP/RAW 264.7-induced differentiation experiment.

B. Gating strategy for analyzing M2 macrophages in the THP-induced differentiation experiment.

C. Gating strategy for assessing M1 and M2 macrophages in subcutaneous tumor tissues and Raw 264.7-induced differentiation experiment.

D. FCM analysis showing that PDCD4 overexpression in BPC inhibits M2 polarization.

E. WB analysis confirmed the knockdown efficiency of eIF4A using siRNA in BPC.

F. FCM analysis showing that inhibition of eIF4A reduced M2 macrophage polarization induced by BPC cells in RAW 264.7 polarization assays.

FCM: Flow Cytometry; **WB**: Western Blot
